# Artificial bioconjugates with naturally occurring linkages: the use of phosphodiester

**DOI:** 10.3762/bjoc.14.169

**Published:** 2018-07-27

**Authors:** Takao Shoji, Hiroki Fukutomi, Yohei Okada, Kazuhiro Chiba

**Affiliations:** 1 Department of Applied Biological Science, Tokyo University of Agriculture and Technology, 3-5-8 Saiwai-cho, Fuchu, Tokyo 183-8509, Japan; 2 Department of Chemical Engineering, Tokyo University of Agriculture and Technology, 2-24-16 Naka-cho, Koganei, Tokyo 184-8588, Japan

**Keywords:** alkyl chain soluble supports, artificial bioconjugates, naturally occurring linkages, 5’-phosphitylation, phosphodiester bonds

## Abstract

Artificial orthogonal bond formations such as the alkyne–azide cycloaddition have enabled selective bioconjugations under mild conditions, yet naturally occurring linkages between native functional groups would be more straightforward to elaborate bioconjugates. Herein, we describe the use of a phosphodiester bond as a versatile option to access various bioconjugates. An opposite activation strategy, involving 5’-phosphitylation of the supported oligonucleotides, has allowed several biomolecules that possess an unactivated alcohol to be directly conjugated. It should be noted that there is no need to pre-install artificial functional groups and undesired and unpredictable perturbations possibly caused by bioconjugation can be minimized.

## Introduction

Peptides and oligonucleotides are of exceptional importance since they are promising pharmaceutical candidates for the treatment of a range of diseases that are beyond traditional small molecule drugs [1–7]. Due to their iterative structures, chemical syntheses can technically be divided into two parts; deprotection and coupling reactions, enabling simple repeated procedures for their production, and bioactivities can potentially be tuned by alteration of their sequences. Nowadays, not only canonical amino acids and/or nucleosides but also artificial building blocks with various functions are available, expanding chemical libraries of peptides and oligonucleotides significantly [8–15]. Furthermore, thus designed and synthesized peptides and oligonucleotides can be conjugated with each other to integrate their bioactivities and/or functions, which has been one of the central topics in the field of chemical biology [16–26]. Since peptide synthesis and oligonucleotide synthesis require different chemistries, such conjugations are typically carried out in the latter stages of the synthesis, otherwise subsequent steps are complicated.

Synthetic chemists, armed with various elegant artificial orthogonal bond formations, including alkyne–azide cycloaddition [27–34], thiol–ene ligation [35–38], Staudinger ligation [39–40], inverse-electron-demand Diels–Alder reaction [41–44], and hydrazone/oxime formation [45–48], have developed selective conjugation reactions under mild conditions. Although these bond-forming reactions have proven to be truly powerful approaches and will remain as first options to create novel bioconjugates, artificial functional groups such as alkynes or azides must be pre-installed into the respective biomolecules. Thus generated linkages are also artificial, possibly causing significant perturbation from their native forms, which generally affect bioactivities negatively. Arguably, naturally occurring linkages that can be formed between native functional groups would be safe alternatives, and are known as natural conjugates such as nucleopeptides and nucleolipids [49].

**Figure 1:**
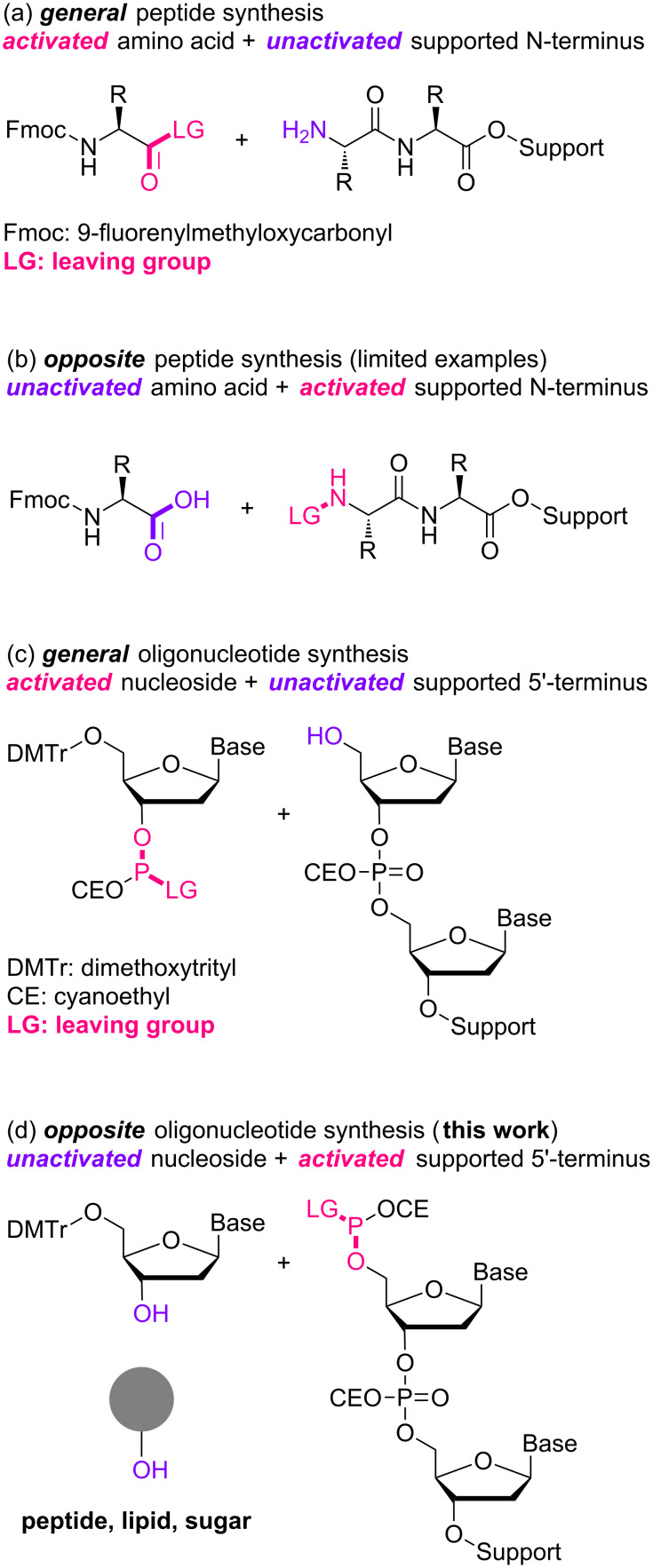
Schematic illustration of possible support-assisted methods.

**Figure 2:**
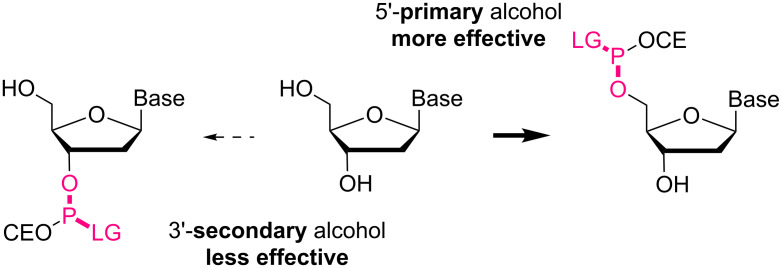
Expected reactivity of 5’- and 3’-terminus for the activation.

We have been developing alkyl chain soluble support (ACSS)-assisted liquid-phase methods, specifically for peptide and oligonucleotide syntheses [50–61]. In both cases, the supported reactants and products are soluble in less-polar solvents, allowing their chemical syntheses even in submolar concentrations. The supported products are readily separated as precipitates by the addition of polar solvents, and washing the precipitates with polar solvents simultaneously rinses away excess amino acids or nucleosides and coupling reagents. We have demonstrated multistep syntheses of up to 28-mers for peptides and 21-mers for oligonucleotides without column purification. In all cases, the C-terminal-activated amino acids or 3’-terminal-activated nucleosides are coupled to the N- or 5’-terminus of the supported reactants via amide or phosphodiester linkages (Figure 1). Such couplings could also be possible in the opposite activating manner. Namely, the activation of the N- or 5’-terminus of the supported reactants would be unique alternatives that allow the use of unactivated amino acids or nucleosides. In peptide synthesis, activation of the N-terminus is rather rare, except for some recent encouraging examples [62–68]; however, this is not the case for oligonucleotide synthesis [69–73]. The activation of the 5’-primary alcohol is expected to be even more effective than that of the 3’-secondary alcohols (Figure 2), and the 5’-primary alcohol could then be coupled not only to other nucleosides but also various alcohols via a naturally occurring phosphodiester linkage. Described herein is a simple and straightforward access to artificial bioconjugates with naturally occurring linkages.

## Results and Discussion

The present work began with the optimization of the reaction conditions for the activation of the 5’-terminus, more specifically, 5’-phosphitylation [74]. The supported trinucleotide **1** was prepared from the support in 83% yield over 8 steps (Scheme S1, Supporting Information File 1) and used as a model in combination with 5-(benzylmercapto)-1*H*-tetrazole (BMT) as an activator (Table 1). The reaction was found to be rather sensitive to the concentration of the starting material (Table 1, entries 1–3). The best result was obtained at 25 mM concentration in dichloromethane (CH_2_Cl_2_). Although tetrahydrofuran (THF) is one of the typical reaction solvents for ACSS-assisted liquid-phase synthesis, this was not the case for the 5’-phosphitylation (Table 1, entry 4). When the activator was switched to tetrazole, the yield was slightly decreased (Table 1, entry 5), while dicyanoimidazole (DCI) was proven to be an inefficient option for the reaction (Table 1, entry 6). It should be noted that the 5’-activated supported trinucleotide **2** was stable throughout the work-up procedure routinely used for the ACSS-assisted liquid-phase method. Namely, the 5’-activated supported trinucleotide **2** was readily separated as a precipitate by the addition of acetonitrile, and washing the precipitate with acetonitrile simultaneously rinses away excess reagents to afford the pure form, which could to be used for further reactions without column purification.

**Table 1:** Optimization of the conditions for the 5’-phosphitylation.

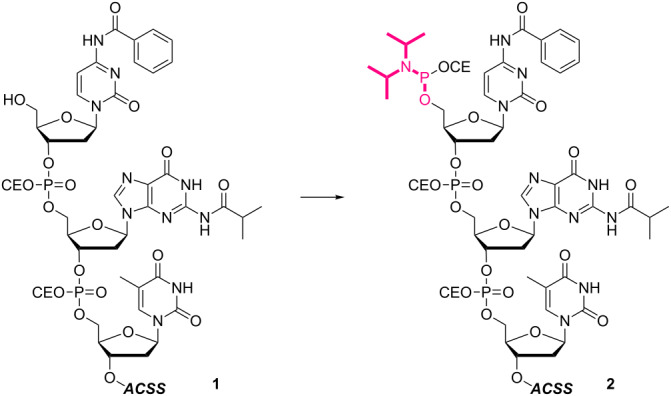				

entry^a^	activator^b^	solvent	concentration (mM)	yield (%)^c,d^

1	BMT	CH_2_Cl_2_	5	61 (24)
2	BMT	CH_2_Cl_2_	25	91 (0)
3	BMT	CH_2_Cl_2_	100	0 (81)
4	BMT	THF	25	0 (86)
5	tetrazole	CH_2_Cl_2_	25	83 (11)
6	DCI	CH_2_Cl_2_	25	40 (46)

^a^Reactions were carried out using 3.0 equiv of 2-cyanoethyl-*N*,*N*,*N'*,*N'*-tetraisopropylphosphorodiamidite in the presence of 3 Å MS at rt for 30 min. ^b^3.0 equiv was used. ^c^Yields were determined by ^31^P NMR analysis. ^d^Recovered starting material was reported in parenthesis.

**Scheme 1:**
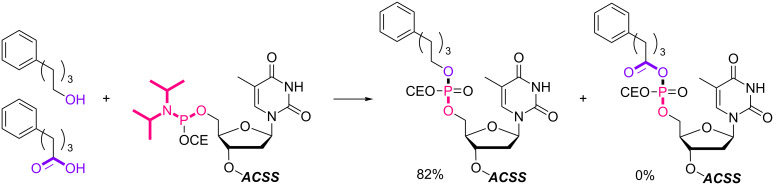
Competition experiment between alcohol and carboxylic acid. Reagents and conditions: (i) 4-phenylbutyl alcohol or 4-phenylbutanoic acid (2.0 equiv), BMT (2.0 equiv), CH_2_Cl_2_/CH_3_CN (10:1, v/v), rt, 30 min; (ii) 0.67% BPO/DMP/CH_2_Cl_2_, rt, 30 min, 82% over 2 steps.

**Scheme 2:**
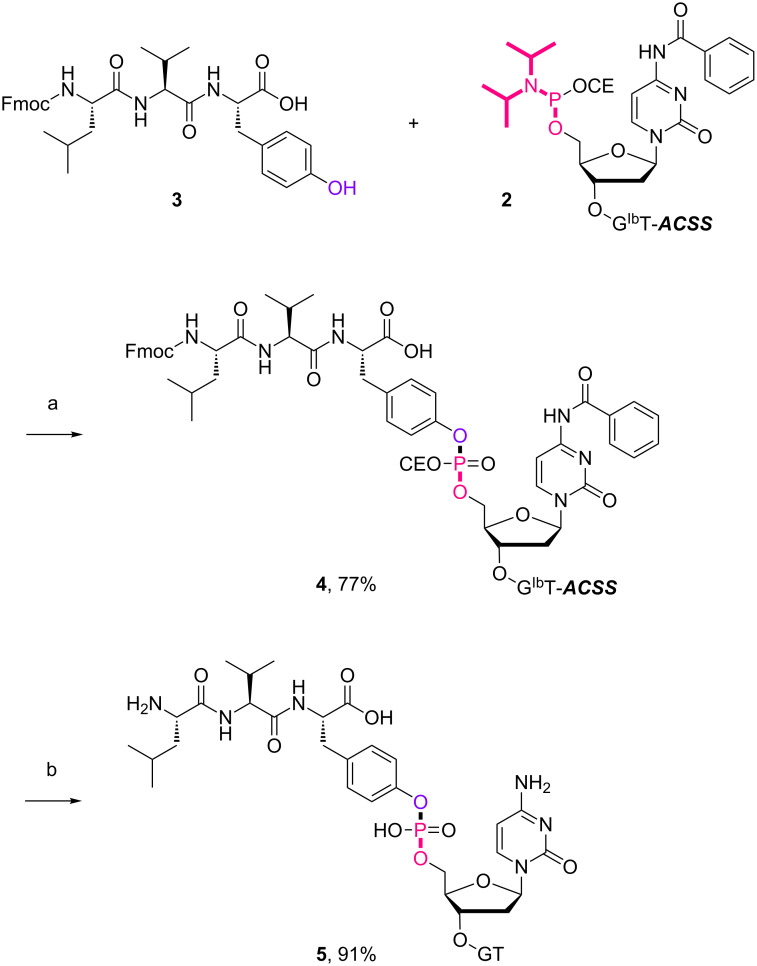
Conjugation between the 5’-activated supported trinucleotide **2** and the tripeptide **3**. Reagents and conditions: (a) (i) tripeptide (2.0 equiv), BMT (2.0 equiv), CH_2_Cl_2_/CH_3_CN/DMF (20:2:1, v/v/v), rt, 30 min; (ii) 0.67% BPO/DMP/CH_2_Cl_2_, rt, 30 min, 77% over 2 steps. (b) (i) 5% DCA/CH_2_Cl_2_, rt, 5 min; (ii) NH_3_ aq/EtOH (3:1, v/v), 70 °C, 3 h; (iii) TEA·3HF/DMF, rt, 24 h, 91% over 3 steps.

**Figure 3:**
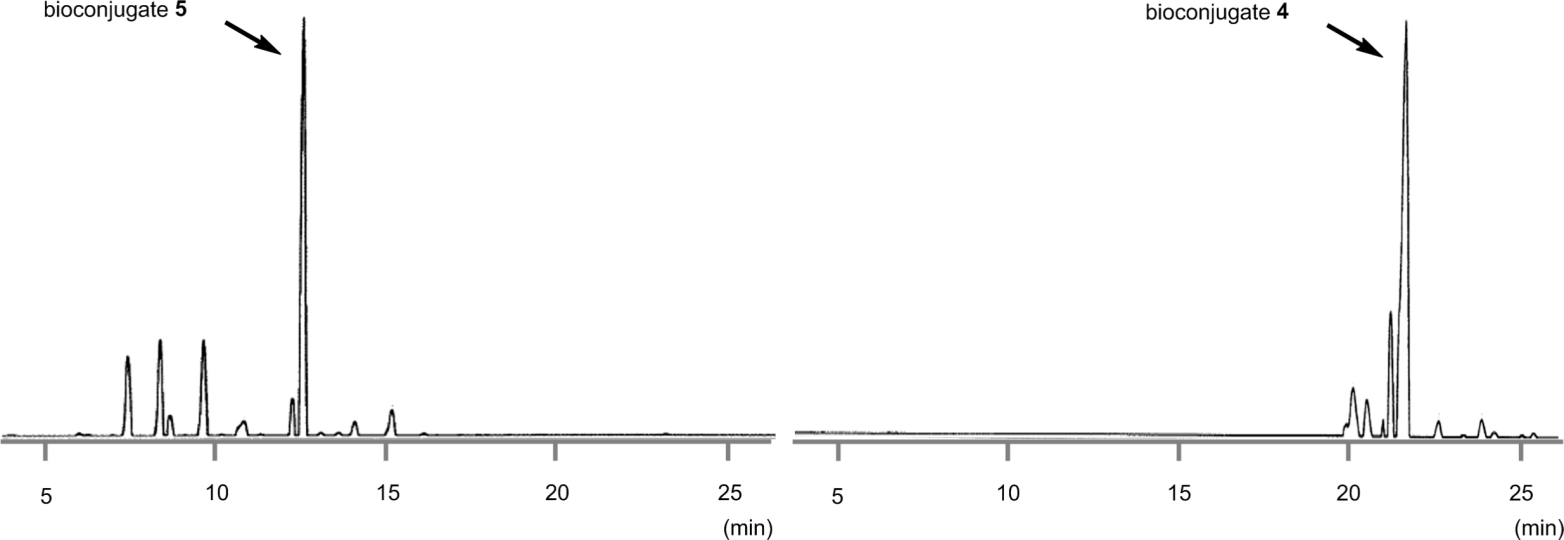
HPLC spectra of the crude protected bioconjugate **4** and the crude deprotected bioconjugate **5**.

With the optimized 5’-phosphitylation conditions in hand, we then investigated conjugation using the 5’-activated supported trinucleotide **2** as a model. As expected, unactivated nucleosides could be coupled to the activated 5’-terminus without difficulty (Scheme S2, Supporting Information File 1). Furthermore, to our delight, the conjugation was compatible with carboxylic acids that are potential nucleophiles for the activated 5’-terminus and can also induce side reactions of the phosphoramidite (Scheme 1). This compatibility is of immense versatility since synthesized peptide fragments, which are cleaved from their supports thus freeing the C-terminus, could directly be conjugated to the 5’-terminus of oligonucleotides via tyrosine, serine, or threonine side chains. In order to demonstrate such a versatile conjugation, tripeptide **3** was prepared from the support in 94% yield over 6 steps (Scheme S3, Supporting Information File 1) and used as a model in combination with the 5’-activated supported trinucleotide **2** (Scheme 2 and Figure 3). To our satisfaction, the conjugation took place smoothly to afford the desired bioconjugation product **4** and basic deprotection gave the native form **5** with a naturally occurring linkage in 70% yield over 5 steps. The impurities can reasonably be assigned as the hydrolized and/or oxidized products of the 5’-activated supported trinucleotide **2** [75].

**Scheme 3:**
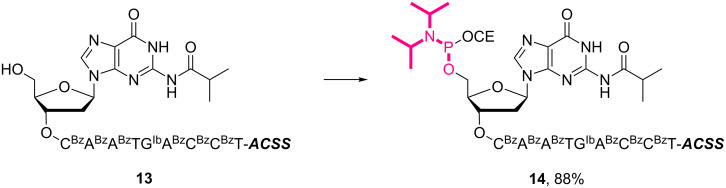
5’-Phosphitylation of supported decanucleotide **13**. Reagents and conditions: 2-cyanoethyl-*N*,*N*,*N'*,*N'*-tetraisopropylphosphorodiamidite (3.0 equiv), BMT (2.0 equiv), CH_2_Cl_2_/CH_3_CN (10:1, v/v), rt, 30 min, 88%.

**Scheme 4:**
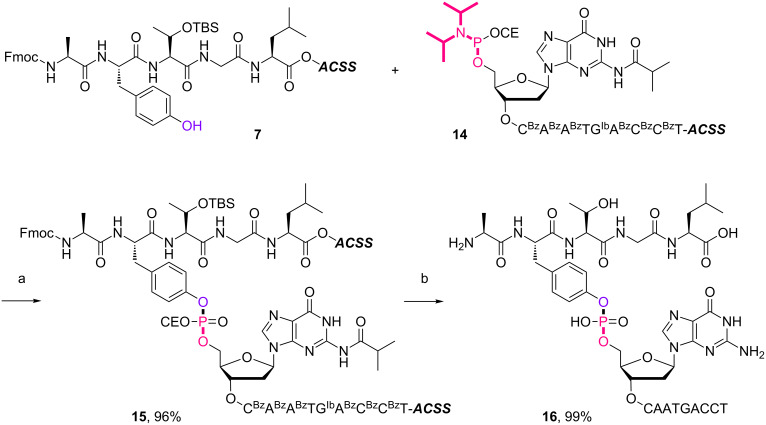
Conjugation between 5’-activated supported decanucleotide **14** and supported pentapeptide **7**. Reagents and conditions: (a) (i) pentapeptide (2.0 equiv), BMT (2.0 equiv), CH_2_Cl_2_/CH_3_CN/DMF (20:2:1, v/v/v), rt, 30 min; (ii) 0.67% BPO/DMP/CH_2_Cl_2_, rt, 30 min, 96% over 2 steps. (b) (i) 5% DCA/CH_2_Cl_2_, rt, 5 min; (ii) NH_3_ aq/EtOH (3:1, v/v), 70 °C, 3 h; (iii) TEA 3HF/DMF, rt, 24 h, 99% over 3 steps.

**Figure 4:**
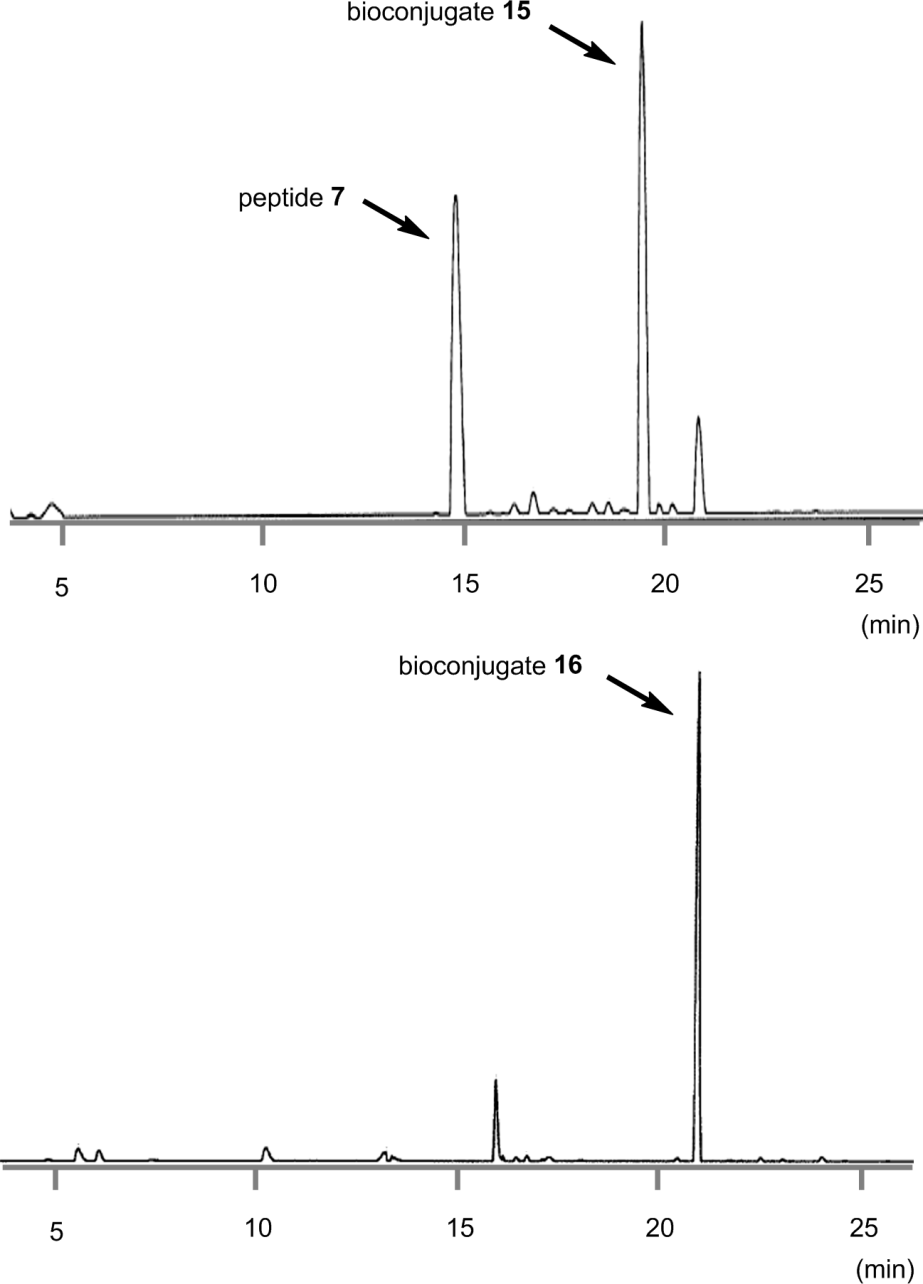
HPLC spectra of the crude protected bioconjugate **15** and the deprotected bioconjugate **16**.

In order to investigate the generality of the current conjugation approach, we then turned our attention to the use of relatively longer peptides, sugars, and lipids as biomolecules (Table 2) [76–77]. When pentapeptide **6** was used instead (see Scheme S4 in Supporting Information File 1 for preparation of pentapeptide **6**), however, the conjugation was not successful at all (Table 2, entry 1). No desired conjugate was obtained under the same reaction conditions that were effective for the conjugation with tripeptide **3**. In this case, no other functional group that could inhibit the conjugation existed in the pentapeptide **6**, and therefore, it could be rationalized that the physical properties, such as solubility, were the issue. This hypothesis was confirmed by the successful conjugation with supported pentapeptide **7**, affording the desired bioconjugate **8** in 67% yield over 2 steps (Table 2, entry 2). It is well-known that peptides with specific sequences can cause aggregation and thus severely inhibit further reactions; however, our support could possibly address such technical problems. Gratifyingly, although further optimization of the reaction conditions is needed for the deprotection reactions (see Schemes S5 and S6 in Supporting Information File 1 for side reactions), both the lipid **9** and the protected sugar **10** were also effectively conjugated to the activated 5’-terminus under the same reaction conditions to give the desired bioconjugates **11** and **12**, respectively (Table 2, entries 3 and 4). We finally examined whether relatively longer supported oligonucleotides were compatible with the methodology disclosed here. Therefore, supported decanucleotide **13** was prepared from the support in 54% yield over 27 steps (Scheme S7, Supporting Information File 1). The results were better than expected, our optimized conditions for the 5’-phosphitylation, conjugation, and deprotection were all even more effective with the supported decanucleotide **13** than with the supported trinucleotide **1** and the desired bioconjugate **16** was obtained in 84% yield over 6 steps (Scheme 3, Scheme 4 and Figure 4).

**Table 2:** Conjugation between the 5’-activated supported trinucleotide **2** and several biomolecules. Reagents and conditions: (a) (i) biomolecule (2.0 equiv), BMT (2.0 equiv), CH_2_Cl_2_/CH_3_CN/DMF (20:2:1, v/v/v), rt, 30 min; (ii) 0.67% BPO/DMP/CH_2_Cl_2_, rt, 30 min. (b) (i) 5% DCA/CH_2_Cl_2_, rt, 5 min; (ii) NH_3_ aq/EtOH (3:1, v/v), 70 °C, 3 h; (iii) TEA 3HF/DMF, rt, 24 h.

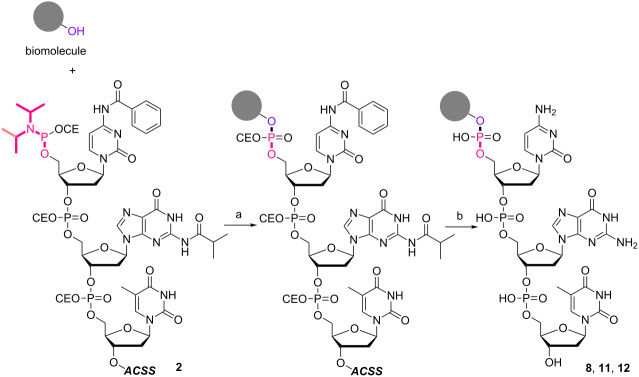				

entry	biomolecule	conjugation yield (%)^a^	bioconjugate	deprotection yield (%)^b^

1	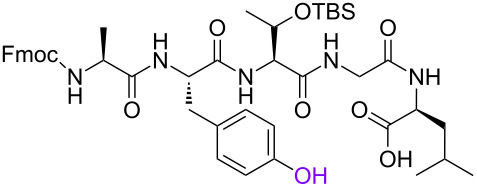 **6**	n.d.	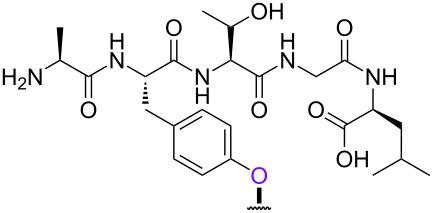 **8**	–
2	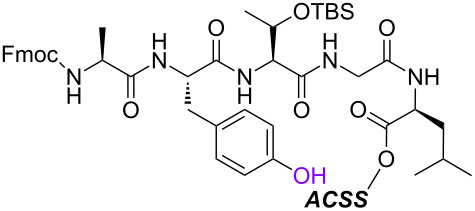 **7**	70	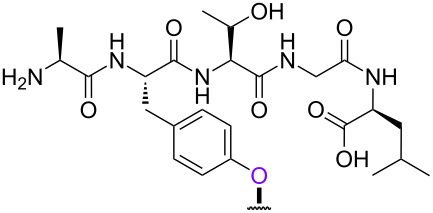 **8**	67
3^c^	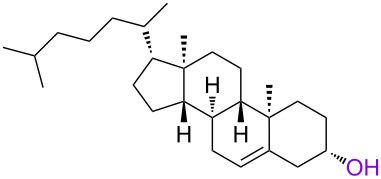 **9**	68	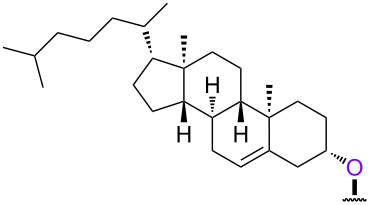 **11**	28
4^c^	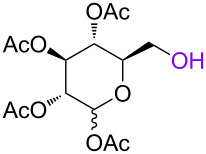 **10**	96	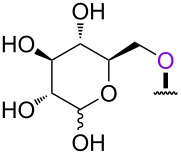 **12**	41

^a^Yields were determined by ^31^P NMR and HPLC analyses. ^b^Yields were determined by HPLC analysis. ^c^CH_2_Cl_2_/CH_3_CN (10:1. v/v) was used as a solvent.

## Conclusion

In conclusion, we have demonstrated that the phosphodiester bond can be an effective linkage not only to construct oligonucleotides but also to conjugate them to various biomolecules, including peptides, sugars, and lipids. The development of a method to activate the supported 5’-terminus, affording useful stable phosphoramidites that are compatible with ACSS-assisted liquid-phase synthesis, has enabled direct conjugation using unactivated alcohols. It should be noted that the thus obtained bioconjugates were all artificial, but constructed via naturally occurring linkages between native functional groups. The approach presented herein should be promising to design and synthesize novel bioconjugates without undesired and unpredictable perturbations possibly derived from artificial linkages.

## Supporting Information

File 1 Additional schemes and figures, general remarks, synthesis and characterization data, including copies of ^1^H and ^13^C NMR.
