# Supplementary material for: Artificial bioconjugates with naturally occurring linkages: the use of phosphodiester
Source: Beilstein J Org Chem. 2018 Jul 27;14:1946–55. doi: 10.3762/bjoc.14.169 (PMC6071721; doi:10.3762/bjoc.14.169)
Supplement: File 1 — Additional schemes and figures, general remarks, synthesis and characterization data, including copies of 1H and 13C NMR. [file Beilstein_J_Org_Chem-14-1946-s001.pdf]

Supporting Information

for

Artificial bioconjugates with naturally occurring linkages: the use of phosphodiester

Takao Shoji[1], Hiroki Fukutomi[1], Yohei Okada[2] and Kazuhiro Chiba[*,1]

Address: [1]Department of Applied Biological Science, Tokyo University of Agriculture and Technology, 3-5-8 Saiwai-cho, Fuchu, Tokyo 183-8509, Japan and [2]Department of Chemical Engineering, Tokyo University of Agriculture and Technology, 2-24-16 Naka-cho, Koganei, Tokyo 184-8588, Japan

Email: Kazuhiro Chiba - chiba@cc.tuat.ac.jp

* Corresponding author

Additional schemes and figures, general remarks, synthesis and characterization data, including copies of ^{1}H and ^{13}C NMR

Table of Contents

1. Additional schemes and figures S2

2. General remarks S13

3. Synthesis and characterization data S15

4. References S30

5. Copies of ^{1}H and ^{13}C NMR spectra S31

1. Additional schemes and figures

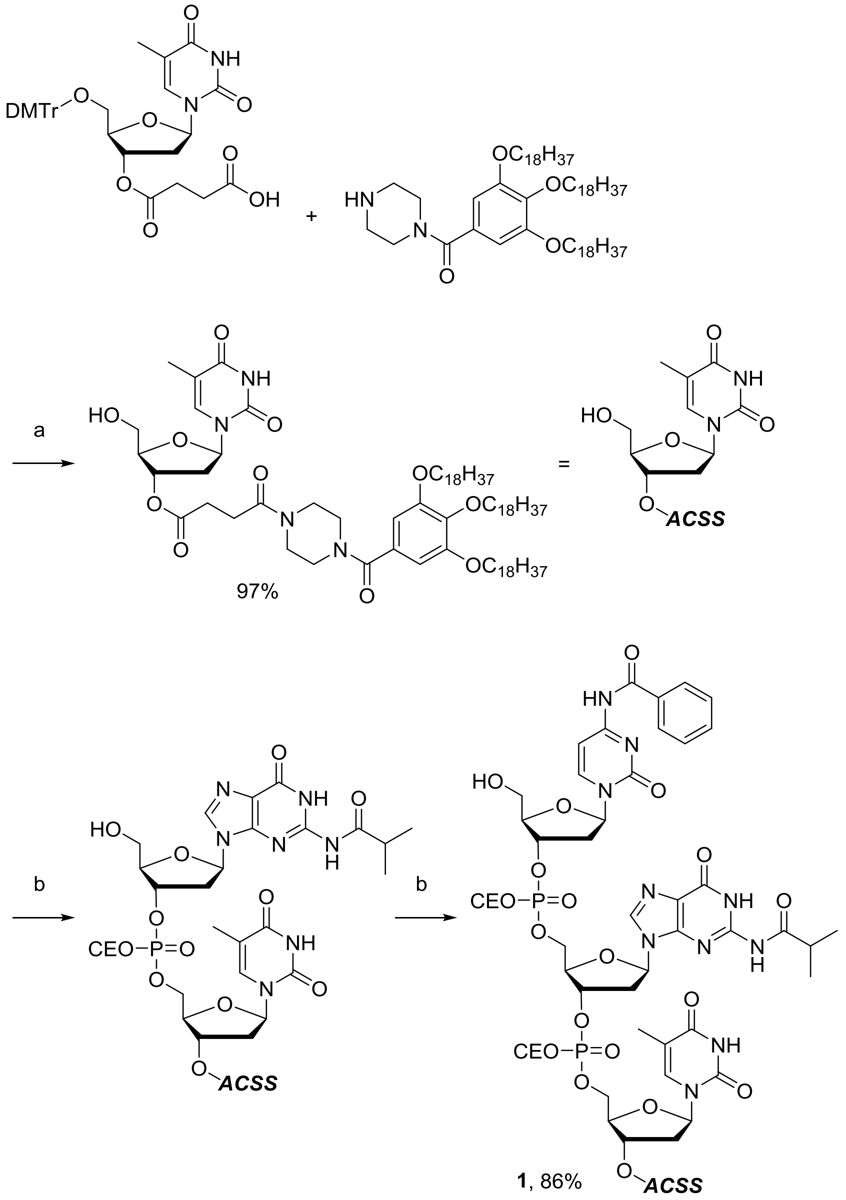

Reagents and conditions: (a) (i) DIPCI (4.0 eq), HOBt (4.0 eq), DIPEA (4.0 eq), CH_2Cl_2, rt, 1h; (ii) 3% DCA/CH_2Cl_2, rt, 5 min, 97% over 2 steps. (b) (i) DMT-Phosphoramidite (2.0 eq), BMT (2.0 eq), CH_2Cl_2/CH_3CN (10:1, v/v), rt, 10 min; (ii) 1% Ac_2O, 1% Pyridine, 1% NMI/CH_2Cl_2, rt, 10 min; (iii) 0.67% BPO/DMP/CH_2Cl_2, rt, 10 min; (iv) 3% DCA/CH_2Cl_2, rt, 5 min, 86% over 6 steps.

Scheme S1: Preparation of the supported trinucleotide **1**.

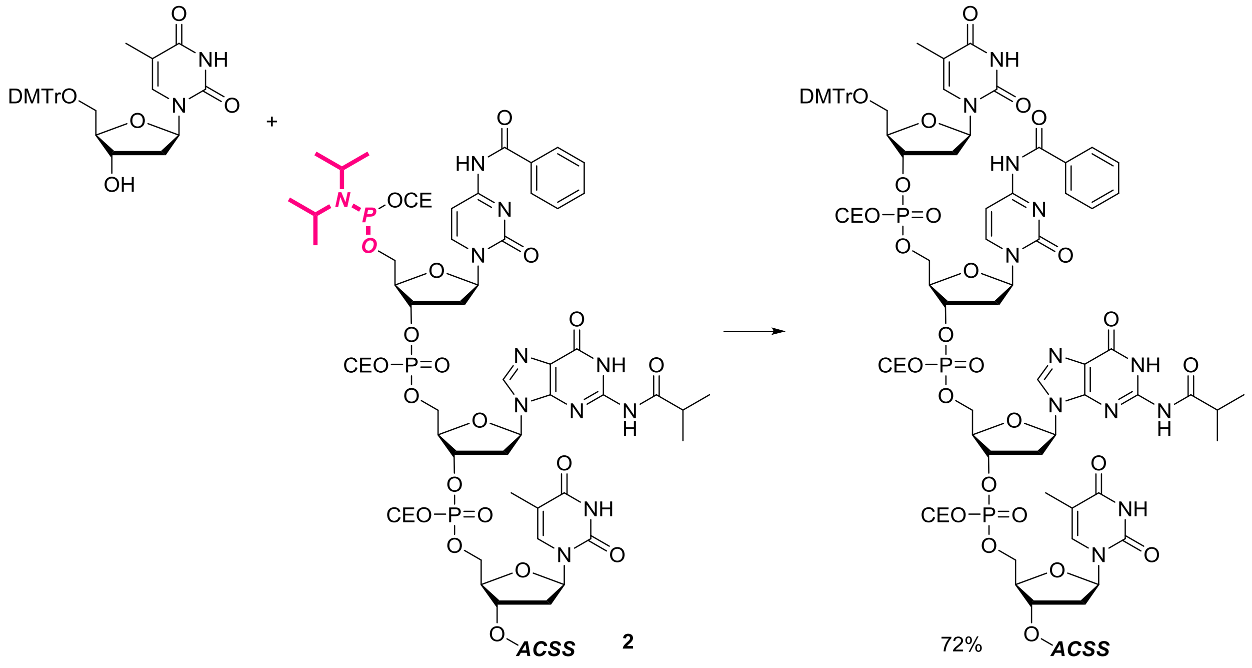

Reagents and conditions: (i) BMT (2.0 eq), CH_2Cl_2/CH_3CN (10:1, v/v), rt, 10 min; (ii) 1% Ac_2O, 1% Pyridine, 1% NMI/CH_2Cl_2, rt, 10 min; (iii) 0.67% BPO/DMP/CH_2Cl_2, rt, 10 min, 72% over 3 steps.

Scheme S2: Conjugation between 5'-activated supported trinucleotide **2** and unactivated DMT-dT.

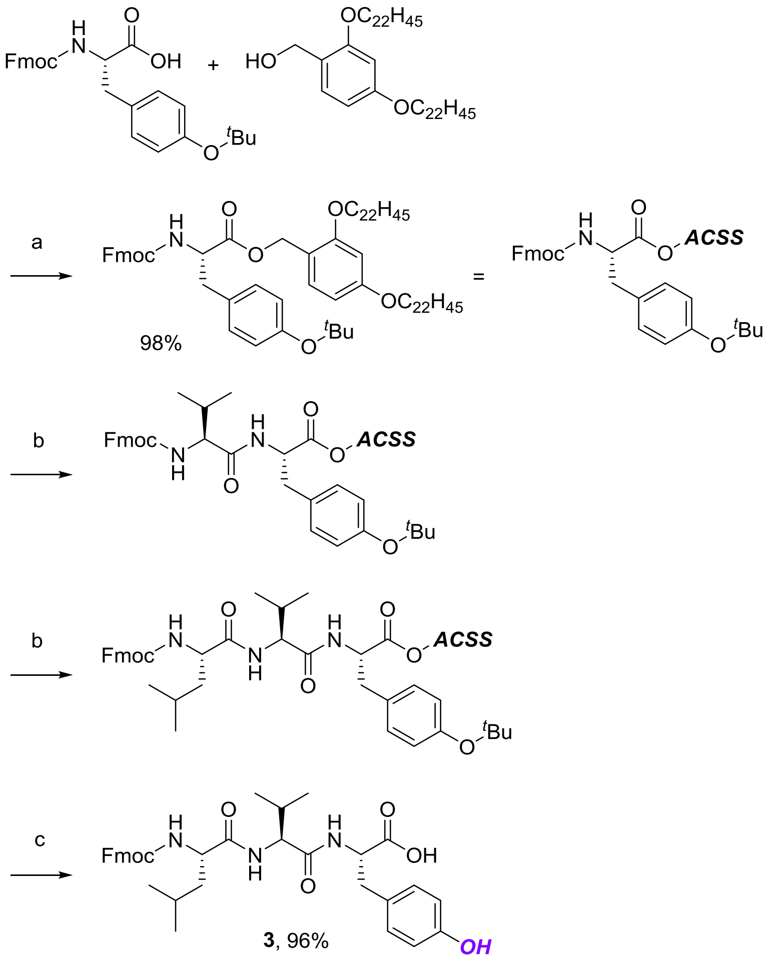

Reagents and conditions: (a) DIPCI (1.8 eq), DMAP (0.1 eq), CH_2Cl_2, rt, 1h, 98%. (b) (i) 3% DBU/THF, rt, 5 min; (ii) Fmoc-AA-OH (1.2 eq), COMU (1.4 eq), DIPEA (1.4 eq), THF, rt, 10 min. (c) 10% TFA/CH_2Cl_2, rt, 5 min, 96% over 5 steps.

Scheme S3: Preparation of the tripeptide **3**.

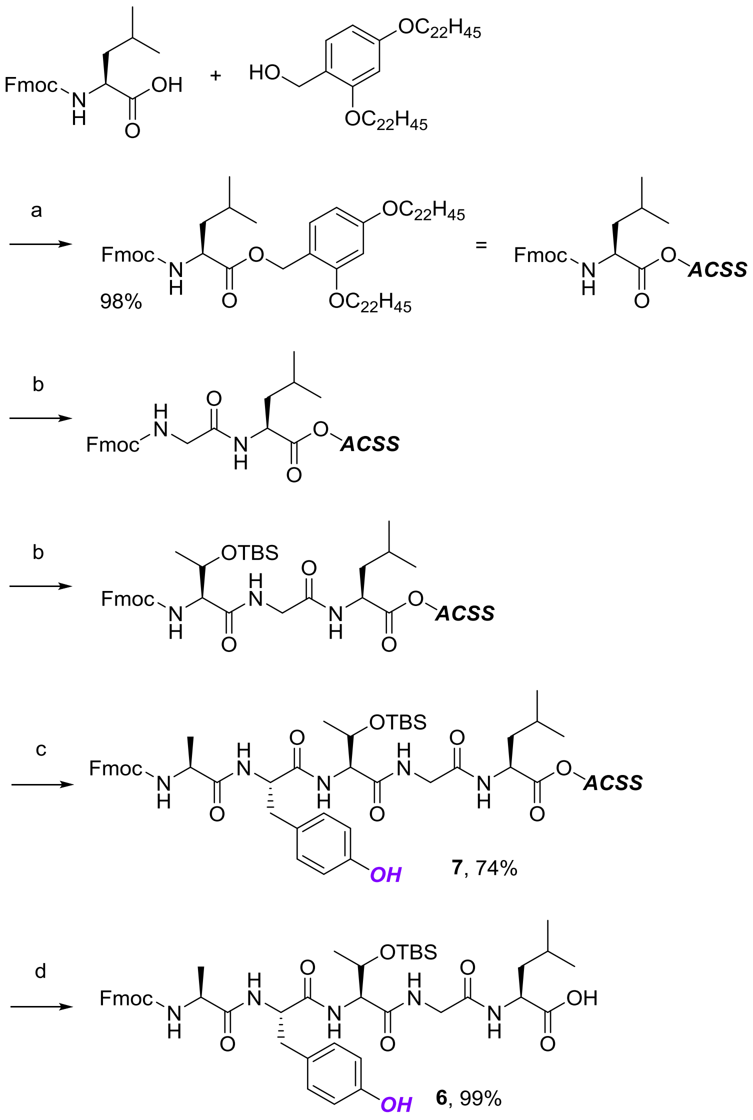

Reagents and conditions: (a) DIPCI (1.8 eq), DMAP (0.1 eq), CH_2Cl_2, rt, 1h, 98%. (b) (i) 3% DBU/THF, rt, 5 min; (ii) Fmoc-AA-OH (1.2 eq), COMU (1.4 eq), DIPEA (1.4 eq), THF, rt, 10 min. (c) (i) 3% DBU/THF, rt, 5 min; (ii) Fmoc-AA-OH (1.2 eq), DMT-MM (1.5 eq), THF/^{i}PrOH (8:2, v/v), rt, 30 min, 74% over 10 steps. (d) 5% DCA/CH_2Cl_2, rt, 5 min, 99%.

Scheme S4: Preparation of the pentapeptide **6**.

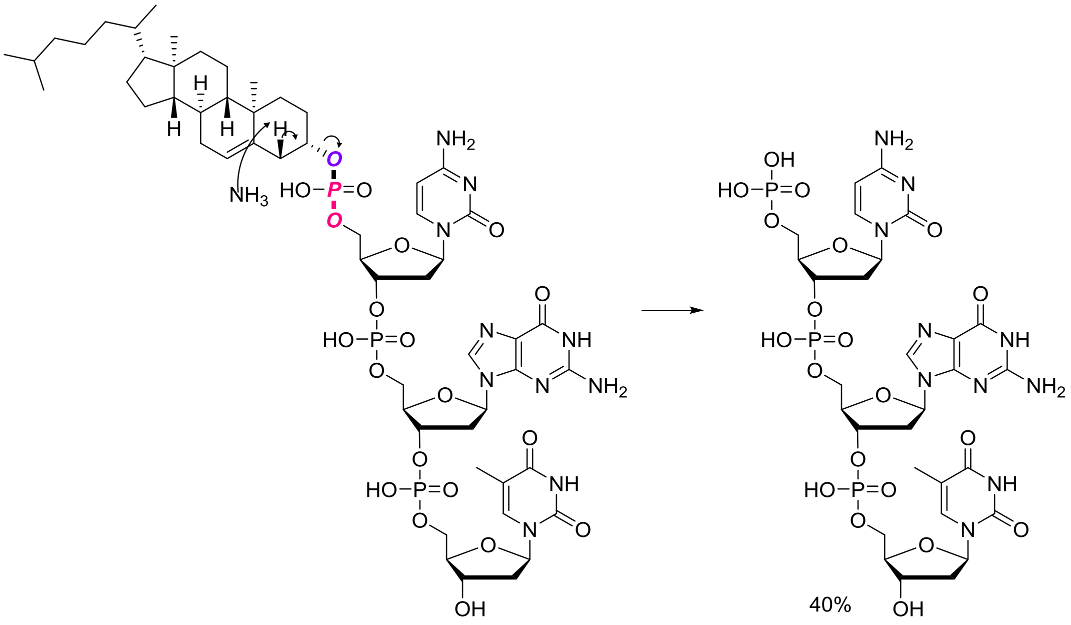

Scheme S5: Side reaction accompanied with the deprotection of the lipid-conjugate.

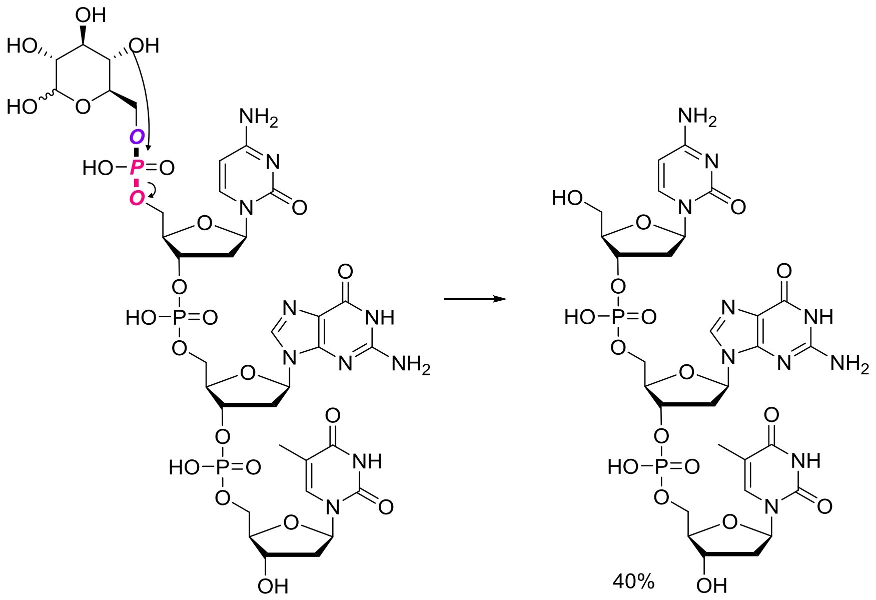

Scheme S6: Side reaction accompanied with the deprotection of the sugar-conjugate.

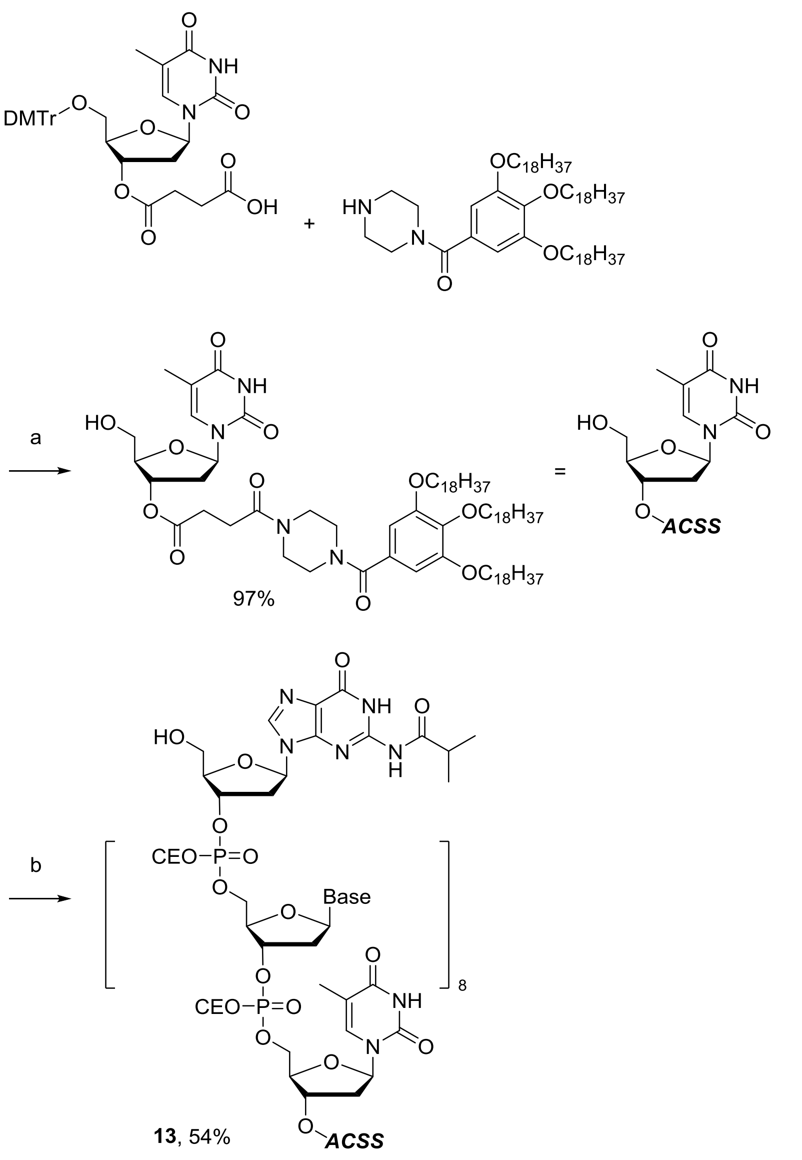

Reagents and conditions: (a) (i) DIPCI (4.0 eq), HOBt (4.0 eq), DIPEA (4.0 eq), CH_2Cl_2, rt, 1h; (ii) 3% DCA/CH_2Cl_2, rt, 5 min, 97% over 2 steps. (b) (i) DMT-Phosphoramidite (2.0 eq), BMT (2.0 eq), CH_2Cl_2/CH_3CN (10:1, v/v), rt, 10 min; (ii) 1% Ac_2O, 1% Pyridine, 1% NMI/CH_2Cl_2, rt, 10 min; (iii) 0.67% BPO/DMP/CH_2Cl_2, rt, 10 min; (iv) 3% DCA/CH_2Cl_2, rt, 5 min, 54% over 27 steps.

Scheme S7: Preparation of the supported decanucleotide (**13**).

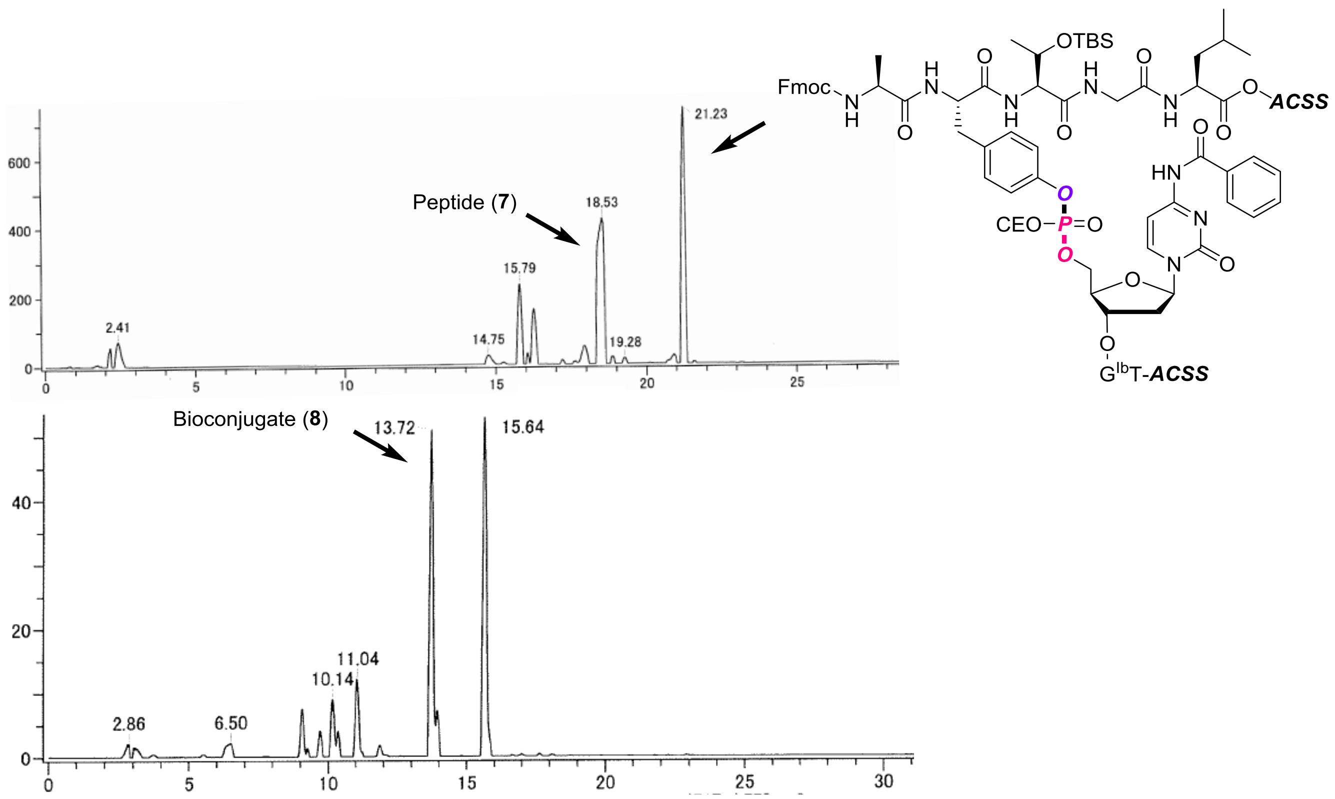

Figure S1: Crude HPLC spectra of protected and deprotected pentapeptide **7**-conjugate.

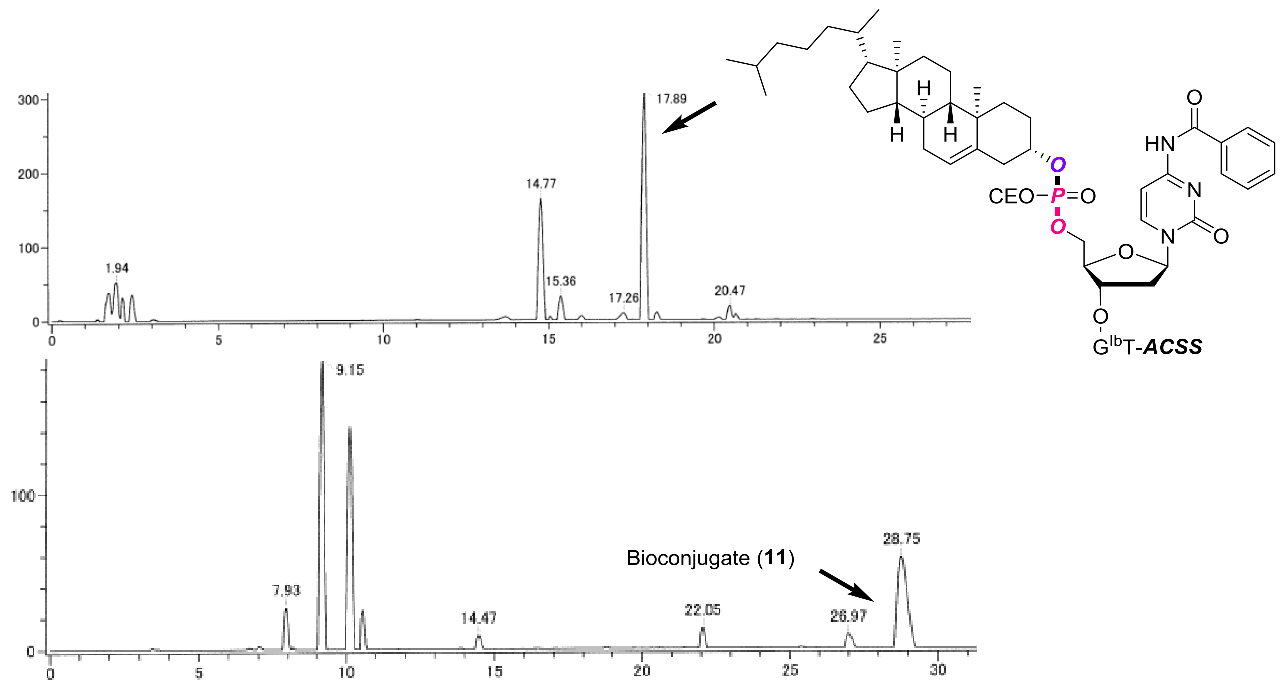

Figure S2: Crude HPLC spectra of protected and deprotected lipid **9**-conjugate.

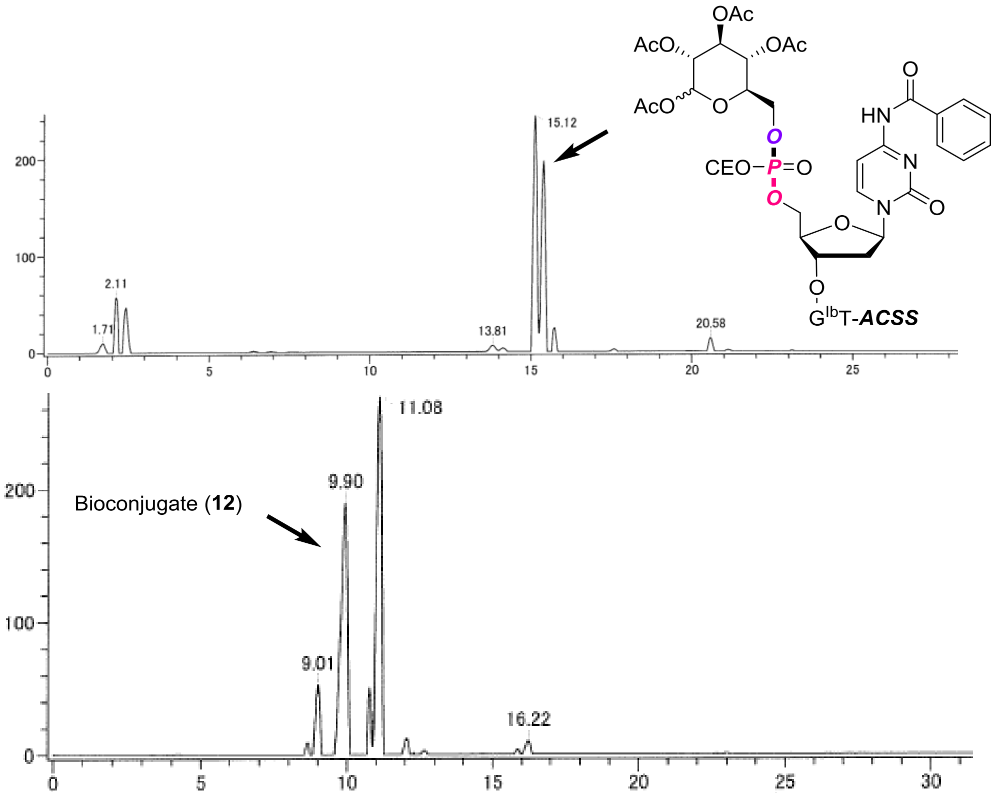

Figure S3: Crude HPLC spectra of protected and deprotected sugar **10**-conjugate.

Bioconjugate **5**

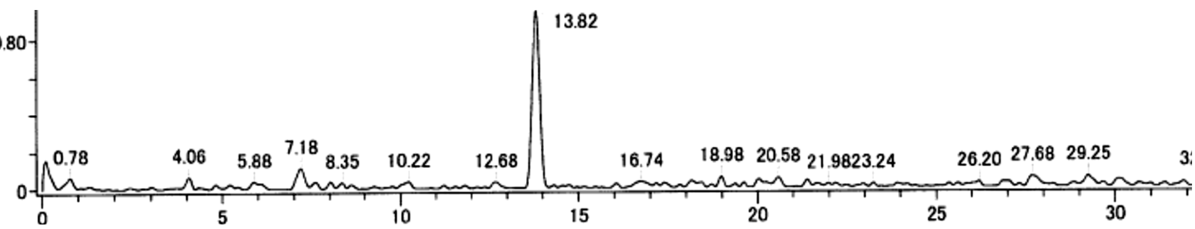

Bioconjugate **8**

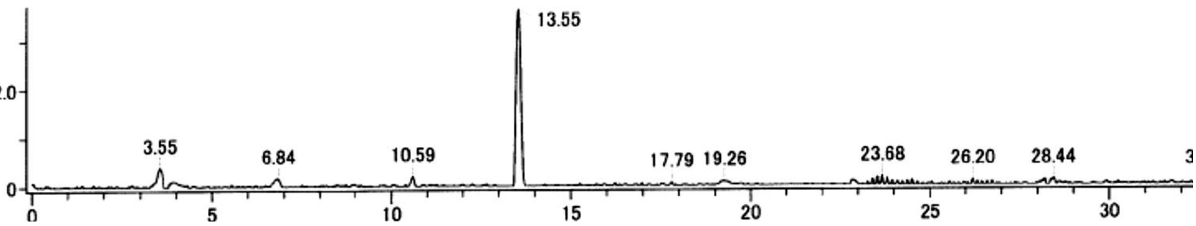

Bioconjugate **11**

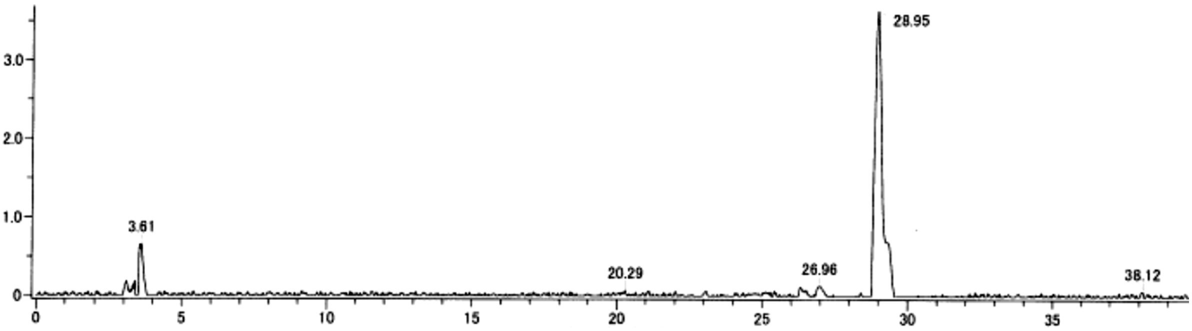

Bioconjugate **12**

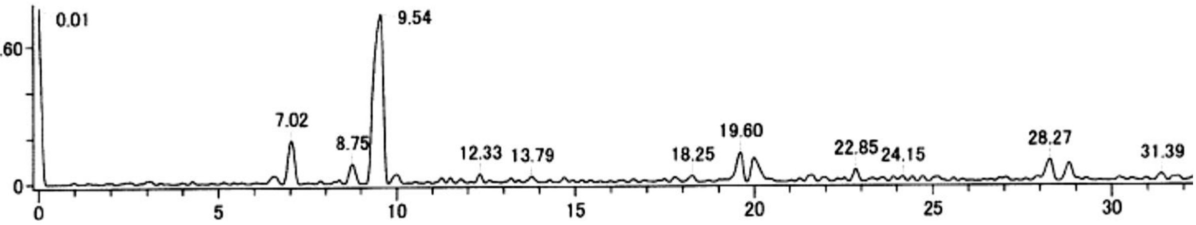

Bioconjugate **16**

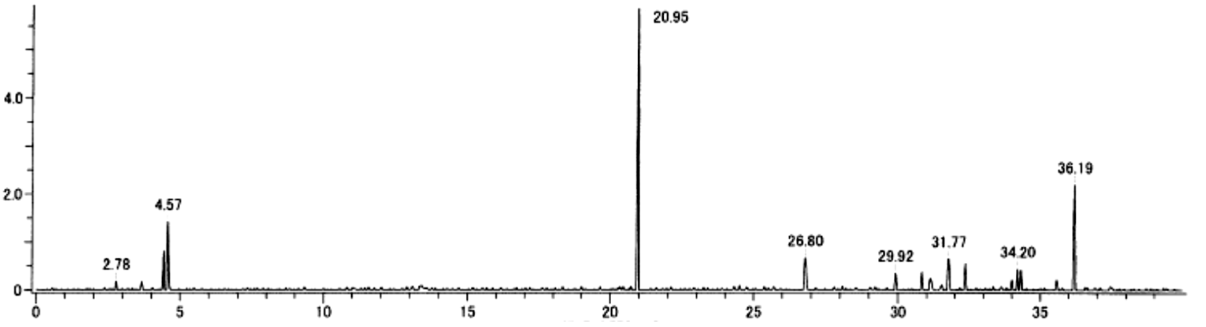

Figure S4: HPLC spectra of deprotected bioconjugates **5**, **8**, **11**, **12**, and **16** after purification.

2. General remarks

All reagents and solvents were purchased from commercial sources and used without further purification. Reactions were monitored by thin-layer chromatography (TLC) carried out on silica gel plates, with detection by UV absorption (254 nm) and by heating the plates after dipping them in a solution of 12 molybdo(VI) phosphoric acid n-hydrate in 95% ethanol. Silica gel (particle size 40−50 μm) was used for column chromatography. ^{1}H NMR spectra were collected on 600 or 400 MHz NMR spectrometers using the deuterated solvent as an internal deuterium reference. Chemical shift data are given in δ units calibrated with residual protic solvent. The multiplicity of a signal is indicated as follows: s, singlet; d, doublet; t, triplet; q, quartet; quin, quintet; m, multiplet. ^{13}C NMR spectra were collected on 150 or 100 MHz spectrometers with proton decoupling using the deuterated solvent as an internal carbon reference. Chemical shift data are given in δ units calibrated with residual solvent. ^{31}P NMR quantitative spectra were collected on 242.95 or 161.83 MHz spectrometers inverse gated proton decoupling. High-resolution mass spectra (HRMS) were collected on electrospray ionization (ESI)- or matrix assisted laser desorption ionization (MALDI)-time-of-flight (TOF) spectrometers.

Abbreviations: BMT, 5’-(benzylmercapto)-1*H*-tetrazole; MS, molecular sieve; dT, 2’-deoxythymidine; dC$^{(Bz)}$, *N*^{4}-benzoyl-2’-deoxycytidine; dG$^{(Ib)}$, *N*^{2}-isobutyryl-2’-deoxyguanosine, dA$^{(Bz)}$, *N*^{6}-benzoyl-2’-deoxyadenosine; NMI, *N*-methylimidazole; BPO, butanone peroxide; DMP, dimethylphthalate; DCA, dichloro acetic acid; THF, tetrahydrofuran; Fmoc, 9-fluorenylmethoxycarbonyl; DIPCI, diisopropylcarbodiimide; DMAP, 4-dimethylaminopyridine; COMU, *N*-[(1-cyano-2-ethoxy-2-oxoethylideneaminooxy)dimethylamino(morpholino)]carbenium hexafluorophosphate; DIPEA, diisopropylethylamine; DMT-MM,

4-(4,6-dimethoxy-1,3,5-triazin-2-yl)-4-methylmorpholinium chloride; DBU, 1,8-diazabicyclo[5.4.0]undec-7-ene; TBDMS, *tert*-butyldimethylsilyl; TFA, tirifluoroactic acid.

3. Synthesis and characterization data

Supported dT (S3)

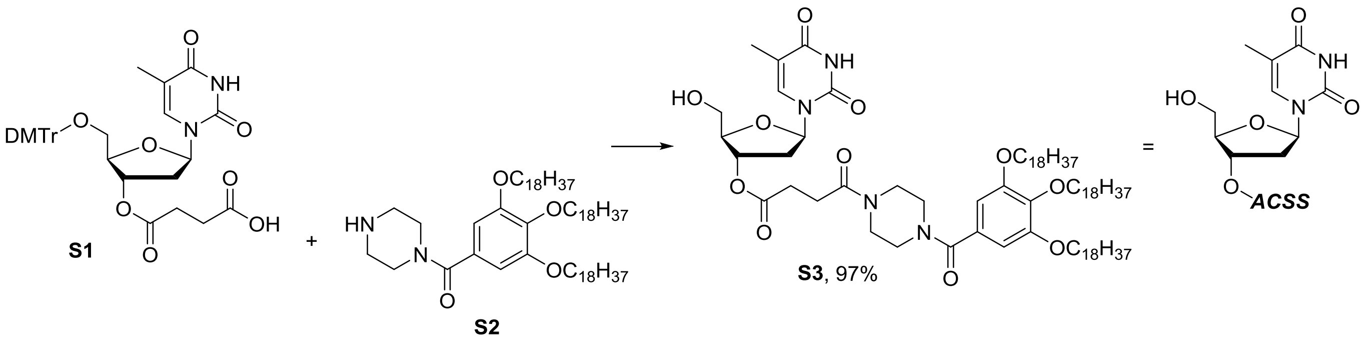

The titled compound was synthesized according to the reported procedure.[1] ^{1}H NMR ($CDCl_3$, 400 MHz) δ 8.066 (0.7H, br), 7.469 (1H, s), 6.576 (2H, s), 6.203 (1H, dd, *J* = 8.25, 6.19 Hz), 5.928 (0.5H, s), 5.36 (1H, ddd, *J* = 6.19, 2.75, 2.75 Hz), 4.112 (1H, dd, *J* = 4.67, 2.75 Hz), 4.011-3.920 (6H, m), 3.921-3.859 (2H, m), 3.825-3.307 (7H, m), 3.196-3.059 (1.5H, m), 2.676 (4H, m), 2.478-2.369 (2H, m), 1.916 (3H, s), 1.821-1.652 (6H, m), 1.519-1.378 (6H, m), 1.365-1.160 (84H, m), 0.866 (9H, t, *J* = 6.87 Hz).

5'-Phosphitylation of the Supported dT

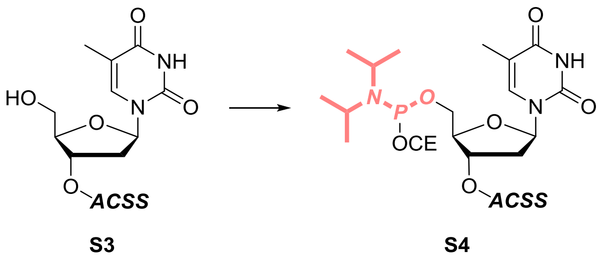

To a solution of the supported dT **S3** (132 mg, 0.10 mmol) in CH_2Cl_2 (4 mL) stirred at r.t. was added BMT (61.8 mg, 0.32 mmol), 3 Å MS, and 2-cyanoethyl-*N*,*N*,*N'*,*N'*-tetraisopropylphosphorodiamidite (101 µL, 0.30 mmol). The resulting reaction mixture was stirred at rt for 20 min, diluted with MeCN, and collected by vacuum filtration. The residue was repeatedly washed with MeCN and dried in vacuo to give the 5'-phosphitylated product **S4** in 93% (141 mg, 0.093 mmol) as a white solid. ^{1}H NMR (600 MHz, $CDCl_3$) δ 8.003 (1H, br), 7.598 (1H, d, *J* = 48.54 Hz), 6.576 (2H, s), 6.334 (1H, ddd, *J* = 33.89, 9.16. 5.04 Hz), 5.337

(1H, dd, *J* = 27.02, 5.95 Hz), 4.201 (1H, d, *J* = 12.36 Hz), 4.018-3.839 (8H, m), 3.821-3,745 (2H, m), 3.730-3.376 (8H, m), 2.726-2.586 (6H, m), 2.422 (1H, dddd, *J* = 16.03, 13.74, 5.50, 4.58), 2.158 (1H, dddd, *J* = 8.93, 7.79, 5.95, 3.21 Hz), 1.960 (3H, d, *J* = 27.02 Hz), 1.812-1.686 (6H, m), 1.576 (2H, s), 1.501-1.383 (6H, m), 1.370-0.955 (96H, m), 0.865 (9H, t, *J* = 6.87 Hz); ^{13}C NMR (150 MHz, $CDCl_3$) δ 172.777, 170.775, 169.774, 163.605, 153.336, 150.447, 139.692, 135.593, 129.853, 117.429, 111.375, 111.079, 105.835, 85.069, 84.640, 84.382, 75.774, 75.515, 73.608, 69.404, 64.198, 63.540, 63.387, 58.772, 58.544, 58.467, 58.258, 43.374, 43.260, 43.145, 37.882, 37.558, 32.009, 30.398, 29.806, 29.606, 29.444, 29.234, 26.183, 24.839, 14.198, 12.663, 12.530; ^{31}P NMR (242.95 MHz, $CDCl_3$) δ 150.191, 149.217, 14.835, 9.691, 8.311; IR ν_{max} (cm^{-1}, KBr) 3509.81, 3182.93, 3071.08, 2912.95, 2851.24, 2360.44, 2338.59, 1696.09, 1640.16, 1577.49, 1470.46, 1426.10, 1380.78, 1324.86, 1273.75, 1217.83, 1200.47, 1155.15, 1110.80, 1048.12, 1031.73, 974.84, 885.17, 716.43, 558.29; HRMS $[M + Na]^+$ calculated for $C_{88}H_{155}N_6O_{12}PNa$ 1542.1333, found 1542.1309.

General procedure for elongation of oligonucleotides

Carried out according to the reported procedure.[1] To a solution of supported oligonucleotide (1.0 mmol) in dichloromethane (88 mL) stirred at rt was added amidite monomer and 0.25 M BMT/MeCN (8.8 mL). The resulting reaction mixture was stirred at rt for 20 min, followed by the addition of 1% Ac_2O, 1% pyridine, and 1% NMI. The resulting reaction mixture was stirred at rt for 10 min, followed by the addition of 0.67% BPO/DMP/CH_2Cl_2. The resulting reaction mixture was stirred at rt for 10 min, diluted with MeOH (100 mL), and collected by vacuum filtration. The residue was repeatedly washed with MeOH and dried in vacuo to give the elongated product as a white solid.

General procedure for deprotection of DMTr

To a solution of supported oligonucleotide (1.0 mmol) in CH_2Cl_2 (20 mL) stirred at rt was added 5% DCA/ CH_2Cl_2 (20 mL). The resulting reaction mixture was stirred at rt for 1 min, diluted with MeOH (100 mL), neutralized with TEA and collected by vacuum filtration. The residue was repeatedly washed with MeOH and dried in vacuo to give the deprotected product as a white solid.

Supported trinucleotide 1, Scheme S1

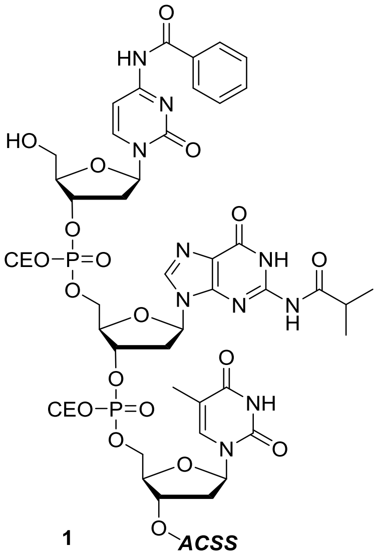

1

White solid. ^{31}P NMR (242.95 MHz, CDCl3) δ -1.624 - -2.680; HRMS [M + Na] calculated for $C_{115}H_{178}N_{14}O_{25}P_2Na$ 2240.2455, found. 2240.2443. Purity was checked by HPLC analysis conditions using a C8 column with H_2O and THF:MeCN=8:2 (70 to 100%, 20 min) as an eluent at flow late of 0.20 mL/min at 50 ^{o}C.

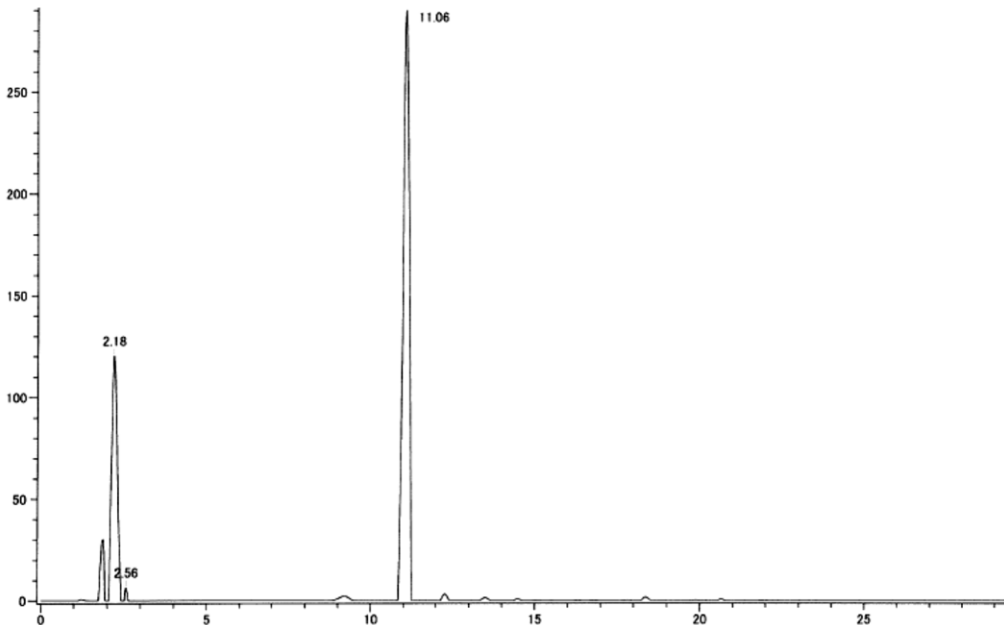

5'-Phosphitylation of the supported trinucleotide 1, see Table 1

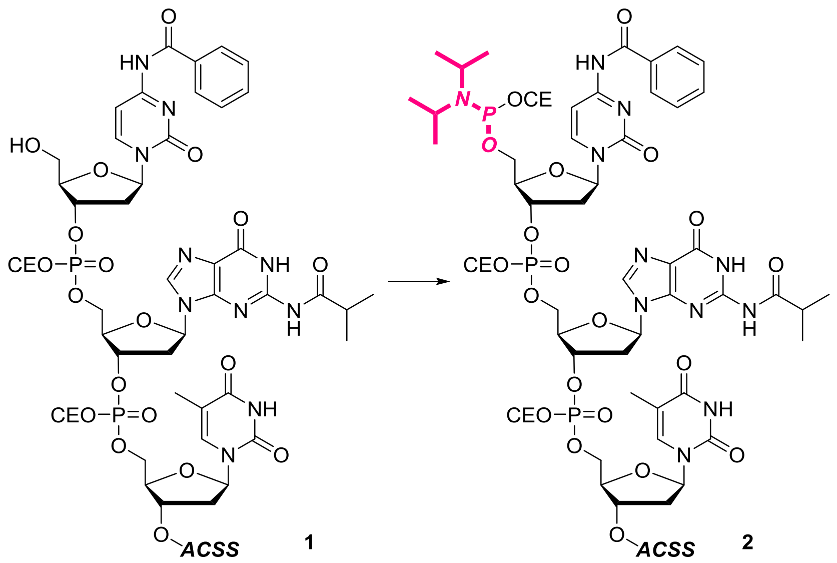

To a solution of the supported trinucleotide **1** (110 mg, 0.050 mmol) in CH_2Cl_2 (2 mL) stirred at rt was added BMT (29.0 mg, 0.15 mmol), 3 Å MS, and 2-cyanoethyl-*N*,*N*,*N'*,*N'*-tetraisopropylphosphorodiamidite (51.0 µL, 0.15 mmol). The resulting reaction mixture was stirred at r.t. for 30 min, diluted with MeCN, and collected by vacuum filtration. The residue was repeatedly washed with MeCN and dried in vacuo to give the 5'-phosphitylated product in 91% (110 mg, 0.045 mmol) as a white solid. ^{31}P NMR (242.95 MHz,

CDCl3) δ 150.1624, 150,083, 149,362, 149.298, 14.781, 9.502, 9.015, -1.624 - -3.329; HRMS $[M + Na]^+$ calculated for $C_{124}H_{195}N_{16}O_{26}P_3Na$ 2240.3534, found 2440.3541.

Competition experiment between alcohol and carboxylic acid, see Scheme 1

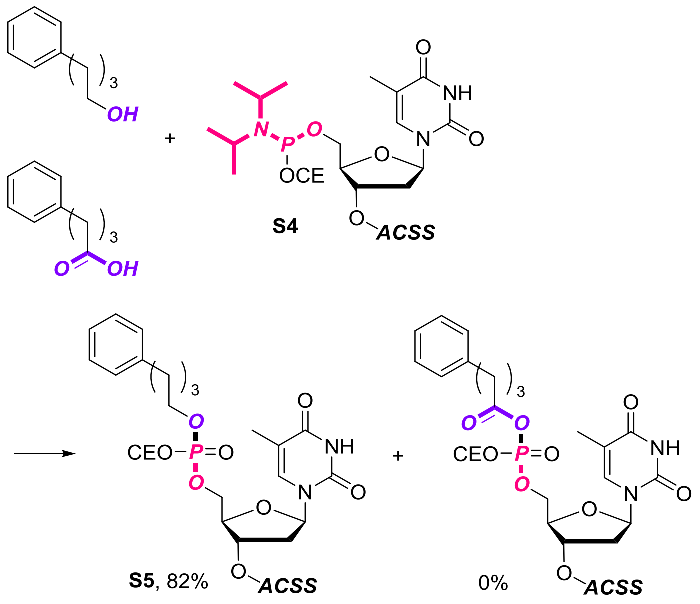

To a solution of the supported dT **S4** (152.0 mg, 0.1 mmol) in CH_2Cl_2/MeCN (10:1, v/v, 4 mL) stirred at rt was added BMT (60 mg, 0.3 mmol), 3 Å MS, 4-phenyl-1-butanol (31 mg, 0.2 mmol), and 4-phenylbutanoic acid (33 mg, 0.2 mmol). The resulting reaction mixture was stirred at rt for 30 min, followed by the addition of 0.67% BPO/DMP/CH_2Cl_2. The resulting reaction mixture was stirred at rt for 30 min, diluted with MeOH (20 mL), and collected by vacuum filtration. The residue was repeatedly washed with MeOH and dried in vacuo to give the desired product in 82% (129.9 mg, 0.082 mmol) as a white solid. ^1H NMR (600 MHz, $CDCl_3$) δ 8.461 (0.2H, br), 7.582-7.376 (1H, m), 7.269-7.241 (2H, m, overlapped on $CDCl_3$), 7.178-7.127 (2H, m), 6.575 (2H, s), 6.377-6.194 (1H, m), 5.372-5.270 (1H, m), 4.421-4.182 (4H, m), 4.135-4.099 (1H, m), 4.019-3.865 (6H, m), 3.823-3.338 (5H, m), 2.801-2.705 (1.5H, m), 2.700-2.548 (5.5H, m), 2.476-2.361 (1H, m), 2.241-2.058 (1H, m), 1.920 (3H, t, J = 10.65 Hz), 1.833-1.745 (4H, m), 1.734-1.630 (6H, m), 1.475-1.413 (6H, m), 1.379-1.241 (87H, m), 1.192 (2H, d, J = 6.19 Hz), 0.864 (9H, t, J = 7.22 Hz); ^{13}C

NMR (150 MHz, $CDCl_3$) δ 172.653, 170.777, 169.676, 163.213, 153.360, 150.258, 142.455, 139.927, 138.998, 135.053, 129.835, 128.495, 126.072, 122.108, 119.571, 116.335, 111.844, 105.917, 84.604, 82.641, 74.340, 73.622, 69.447, 68.844, 676.551, 62.141, 37.094, 35.265, 32.000, 30.401, 29.941, 29.798, 29.444, 29.242, 29.166, 29.108, 27.979, 27.203, 26.179, 22.760, 19.840, 14.239, 12.506; ^{31}P NMR (242.95 MHz, $CDCl_3$) δ -0.785, -1.083; IR ν_{max} (cm^{-1}, KBr) 3414.35, 3211.86, 3071.08, 2912.95, 2851.24, 2349.84, 2248.59, 1701.87, 1640.16, 1583.27, 1465.63, 1431.89, 1386.57, 1329.68, 1267.97, 1245.79, 1222.65, 1160.94, 1121.40, 997.98, 766.57, 716.43, 699.07, 671.11, 552.51; HRMS [M + Na] calculated for $C_{92}H_{154}N_5O_{14}PNa$ 1607.1122, found 1607.1117.

Supported Tyr(t-Bu) (S7)

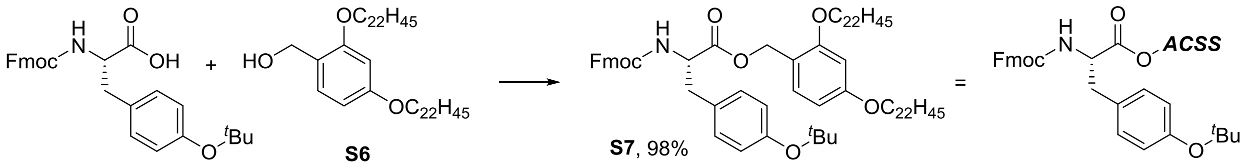

To a solution of Fmoc-Tyr(*t*-Bu)-OH (1.65 g, 3.6 mmol) and compound **S6** (1.5 g, 2.0 mmol) in CH_2Cl_2 (50 mL) stirred at rt was added DIPCI (1.6 mL, 3.6 mmol) and DMAP (180 μL, 0.4 mmol). The resulting reaction mixture was stirred at rt for 2 h, diluted with MeCN (100 mL), and collected by vacuum filtration. The residue was repeatedly washed with MeCN and dried in vacuo to give the titled compound **S7** in 98% yield (2.35 g, 1.96 mmol) as white solid; ^{1}H NMR (600 MHz, $CDCl_3$) δ 7.755 (2H, d, J = 7.56 Hz), 7.559 (2H, d, J = 7.56 Hz), 7.390 (2H, t, J = 7.56 Hz), 7.296 (2H, t, J = 7.56 Hz), 7.161 (1H, d, J = 8.25 Hz), 6.898 (2H, d, J = 8.25 Hz), 6.816 (2H, d, J = 8.25 Hz), 6.436 (1H, s), 6.410 (1H, d, J = 8.25 Hz), 5.274 (1H, d J = 8.25 Hz), 5.152 (2H, dd, J = 23.37, 11.68 Hz), 4.652 (1H, dd, J = 13.75, 5.50 Hz), 4.394 (1H, dd, J = 10.65, 7.56 Hz), 4.311 (1H, dd, J = 10.65, 7.56 Hz), 4.188 (1H, t, J = 7.56 Hz), 3.933 (4H, dd, J = 13.75, 6.87 Hz),

3.061 (2H, ddd, J = 34.02, 14.43, 5.50 Hz), 1.833-1.693 (4H, m), 1.465-1.378 (4H, m), 1.379-1.192 (81H, m), 0.873 (6H, t, J = 7.56 Hz); ^{13}C NMR (150 MHz, $CDCl_3$) δ 171.543, 161.116, 158.703, 155.562, 154.452, 144.025, 143.872, 141.382, 131.817, 130.544, 129.998, 127.767, 127.144, 125.239, 125.201, 124.138, 120.030, 116.449, 115.827, 104.567, 99.713, 78.371, 68.260, 68.202, 66.996, 63.070, 54.845, 47.262, 32.029, 29.807, 29.760, 29.721, 29.530, 29.463, 29.377, 28.926, 26.159, 22.799, 14.229, 2.002; IR ν_{max} (cm^{-1}, KBr) 3330.46, 2911.99, 2861.84, 2638.14, 2465.54, 2403.83, 2319.95, 2281.38, 2253.41, 2019.10, 1895.68, 1733.69, 1617.02, 1594.84, 1510.95, 1460.81, 1382.71, 1337.39, 1293.04, 1259.29, 1181.19, 1136.83, 1036.55, 918.91, 896.74, 830.21, 780.07, 757.89, 740.53, 718.35, 640.25, 612.29, 573.72, 545.76, 500.44, 461.87, 416.55; HRMS $[M + Na]^+$ calculated for $C_{79}H_{123}NO_7Na$ 1220.9192, found 1220.9187.

General procedure for coupling of peptide

To a solution of supported peptide (1.0 mmol) and Fmoc-AA-OH in THF (20 mL) stirred at rt was added COMU (1.4 mmol) and DIPEA (1.4 mmol). The resulting reaction mixture was stirred at rt for 10 min, diluted with MeCN (60 mL), and collected by vacuum filtration. The residue was repeatedly washed with MeCN and dried in vacuo to give the coupled product as a white solid.

General procedure for deprotection of Fmoc

To a solution of supported peptide (1.0 mmol) in THF (19.4 mL) stirred at rt was added DBU (600 µL). The resulting reaction mixture was stirred at rt for 5 min, diluted with MeCN (60 mL), neutralized 1 N HCl (4 mL) and collected by vacuum filtration. The residue was repeatedly washed with MeCN and dried in vacuo to give the deprotected product as a white solid.

Tripeptide 3, Scheme S3

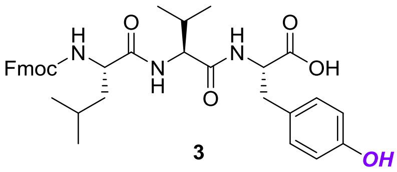

3

95% (from **S7** over 5 steps) as white solid. ^{1}H NMR (600 MHz, CD_3OD) δ 8.24 (0.5H, m), 7.82 (1H, m), 7.77 (2H, m), 7.63 (2H, m), 7.36 (2H, m), 7.28 (2H, m), 6.99 (2H, m), 6.65 (2H, m), 4.55 (1H, m), 4.34 (2H, m), 4.18-4.17 (3H, m), 3.04-3.02 (1H, m), 2.87-2.85 (1H, m), 1.99 (1H, m), 1.61 (1H, m), 1.51-1.45 (2H, m), 0.92-0.86 (12H, m); ^{13}C NMR (150 MHz, CD_3OD) δ 173.96, 173.10, 155.95, 144.06, 143.76, 141.26, 129.96, 127.42, 126.85, 124.87, 119.56, 114.86, 66.34, 58.45, 53.92, 53.64, 40.55, 36.25, 31.01, 24.54, 22.19, 20.50, 18.35, 17.29 ;IR ν_{max} (cm^{-1}, KBr) 3290.93, 3068.19, 2962.13, 2872.45, 1717.30, 1672.95, 1639.20, 1538.92, 1521.56, 1455.03, 1399.10, 1365.35, 1332.57, 1293.04, 1259.29, 1226.5, 1170.58, 1108.87, 1030.77, 986.41, 930.49, 830.21, 734.75, 684.61, 595.90, 528.40; HRMS $[M + Na]^+$ calculated for $C_{35}H_{41}N_3O_7Na$ 638.2837, found 638.2829.

Pentapeptide 7 in Figure S2

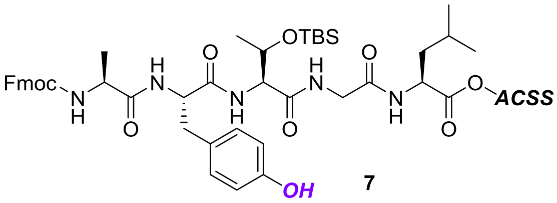

7

74% (from **S6** over 10 steps) as white solid. ^{1}H NMR (600 MHz, $CDCl_3$) δ 7.759 (2H, d, J = 7.33 Hz), 7.578 (2H, d, J = 7.33 Hz), 7.296 (2H, t, J = 7.33 Hz), 7.310 (2H, t, J = 7.33 Hz), 7.190 (1H, d, J = 8.70 Hz),7.031 (2.5H, d, J = 8.24 Hz), 6.703 (2.5H, d, J = 8.24 Hz), 6.628 (1H, d, J = 5.50 Hz), 6.419-6.399 (2H, m), 6.208

(1H, d, J = 6.41 Hz), 5.765 (1H, br), 5.288 (1H, br), 5.149 (2H, dd, J = 41.67, 11,91 Hz), 4.568-4.485 (2H, m), 4.436-4.315 (3H, m), 4.203 (3H, t, J = 6.41 Hz), 3.996 (1H, dd, J = 16.03, 6.41 Hz), 3.919 (4H, t, J = 6.87 Hz), 3.751-3.718 (5H, m), 3.534-3.459 (1H, m), 3.073 (1H, dd, J = 13.28,5.95 Hz), 2.877-2.819 (1H, m), 1.841 (4H, t, J = 6.41 Hz), 1.753 (4H, ddd, J = 15.11, 7.33, 6.41 Hz), 1.655-1.498 (13H, m), 1.464-1.394 (4H, m), 1.365-1.179 (71H, m), 1.006 (2H, d, J = 5.95 Hz), 0.924-0.849 (12H, m), 0.814 (8H, s), 0.029 (3H, s), -0.044 (3H, s); ^{13}C NMR (150 MHz, $CDCl_3$) δ 171.281, 169.402, 160.974, 158.523, 141.437, 134.868, 130.329, 127.870, 127.212, 127.183, 125.085, 123.455, 120.108, 116.809, 104.519, 99.723, 68.212, 68.059, 63.235, 58.715, 51.297, 47.188, 43.412, 41.095, 39.295, 38.473, 34.164, 32.009, 29.787, 29.740, 29.692, 29.511, 29.463, 29.444, 29.358, 29.234, 26.145, 26.097, 25.773, 25.697, 24.906, 22.903, 22.770, 21.912, 17.907, 14.198, 11.471, 10.108, 6.142, -4.499, -5.119; IR ν_{max} (cm^{-1}, KBr) 3295.75, 3070.08, 2918.73, 2851.24, 2462.65, 2360.44, 2338.27, 2248.59, 1696.09, 1640.16, 1617.02, 1589.06, 1515.78, 1465.63, 1448.28, 1375.00, 1324.86, 1256.40, 1184.08, 1155.15, 1121.40, 1087.66, 1043.30, 980.63, 941.09, 834.06, 778.14, 738.60, 716.43, 665.32, 541.90; HRMS [M + Na]$^+$ calculated for $C_{96}H_{155}N_5O_{12}SiNa$ 1622.1367, found 1622.1362. Purity was checked by HPLC analysis using a C8 column with H_2O and THF:MeCN=8:2 (70 to 100%, 20 min) as an eluent at flow late of 0.20 mL/min at 50 ^{o}C.

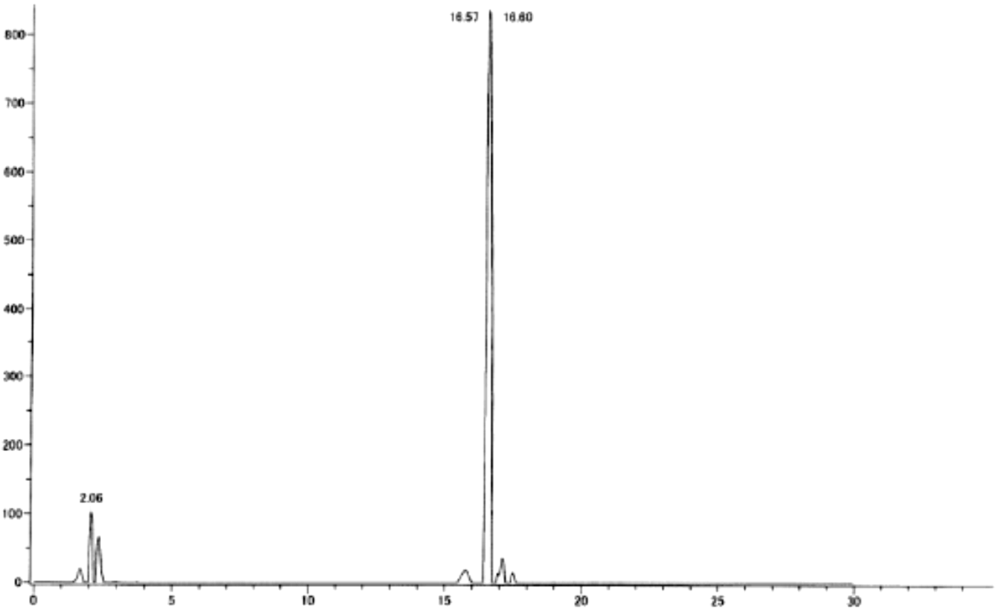

Pentapeptide 6 in Figure S2

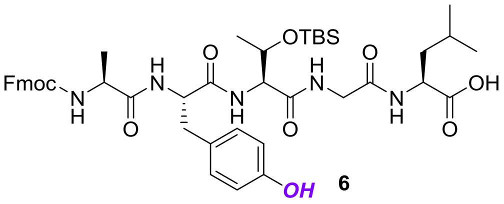

6

99% (from **7** over 1 step) as white solid. HRMS $[M + Na]^+$ calculated for $C_{45}H_{61}N_5O_{10}SiNa$ 882.4080, found 882.4107. Purity was checked by HPLC analysis using a C18 column with 0.1% TFA/H_2O and 0.1% TFA/MeCN (20 to 100%, 20 min) as an eluent at flow late of 1.0 mL/min at 40 ^oC.

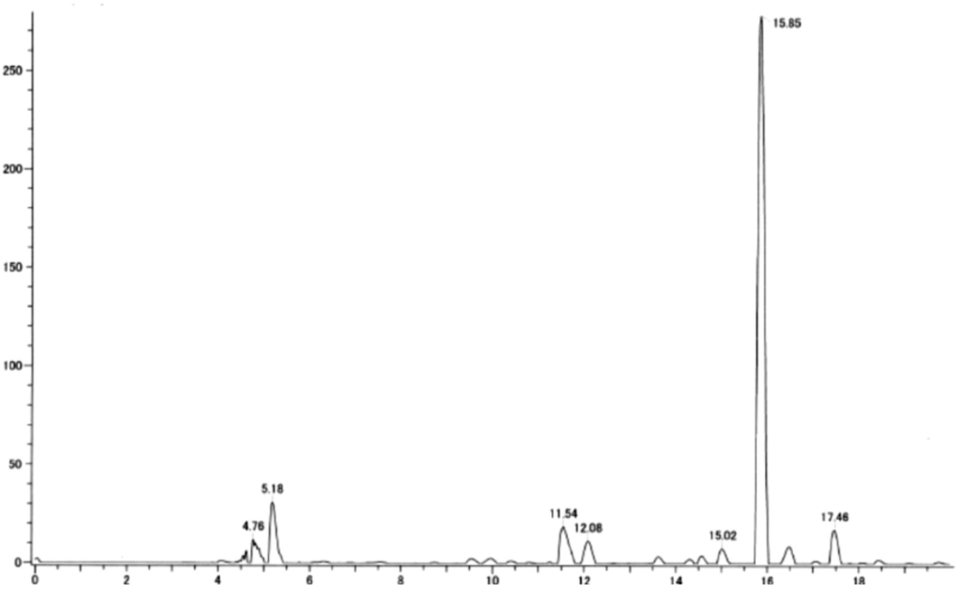

Bioconjugations and deprotection, see Scheme 4 and Table 2

A) Bioconjugations of tripeptide **3** and pentapeptide **6** to 5'-activated supported trinucleotide **2**

To a solution of 5'-activated supported trinucleotide **2** (0.025 mmol) in CH_2Cl_2 (10:1, v/v, 2 mL) stirred at rt was add 5 M peptide **3** or **6**/DMF (100 µL) and 0.25 M BMT/MeCN (200 µL). The resulting reaction mixture was stirred at rt for 30 min, followed by the addition of 0.67% BPO/DMP/CH_2Cl_2. The resulting reaction mixture was stirred at rt for 30 min, diluted with MeOH (10 mL), and collected by vacuum filtration. The residue was repeatedly washed with MeOH and dried in vacuo to give the desired bioconjugate as a white solid.

B) Bioconjugation of supported pentapeptide (**7**) to 5'-activated supported trinucleotide **2**

To a solution of 5'-activated supported trinucleotide **2** (0.025 mmol) in CH_2Cl_2 (10:1, v/v, 6.4 mL) stirred at rt was add supported pentapeptide **7** and 0.75 M BMT/MeCN (200 µL). The resulting reaction mixture was stirred at rt for 30 min, followed by the addition of 0.67% BPO/DMP/CH_2Cl_2. The resulting reaction mixture was stirred at rt for 30 min, diluted with MeOH (30 mL), and collected by vacuum filtration. The residue was repeatedly washed with MeOH and dried in vacuo to give the desired bioconjugate as a white solid.

C) Bioconjugation of lipid **9** and suger **10** to 5'-activated suppored trinucleotide **2**

To a solution of 5'-activated supported trinucleotide **2** (0.025 mmol) in CH_2Cl_2 (10:1, v/v, 2 mL) stirred at rt was add lipid **9** or sugar **10** and 0.25 M BMT/MeCN (200 µL). The resulting reaction mixture was stirred at

r. for 30 min, followed by the addition of 0.67% BPO/DMP/CH_2Cl_2. The resulting reaction mixture was stirred at rt for 30 min, diluted with MeOH (10 mL), and collected by vacuum filtration. The residue was repeatedly washed with MeOH and dried in vacuo to give the desired bioconjugates as white solids.

D) Deprotection and cleavage of ACSS in tripeptide **3** conjugate

Protected tripeptide conjugates (5 μmol) was added to 28% NH_3 aq/EtOH (3:1, v/v). The resulting reaction mixture was stirred at 70 ^{o}C for 3 h, followed by evaporation. The residue was diluted with 50 mM AcOH/NH_3 buffer and purified by C18 column by using 20% MeCN/50 mM AcOH/NH_3 buffer as eluting solvent. The crude mixture was dried by lyophilization. The resulting crude mixture was purified by HPLC to give the desired bioconjugate **5**.

E) Deprotection and cleavage of ACSS in lipid **9** or sugar **10** conjugates

Protected lipid or sugar conjugates (5 μmol) was added to 28% NH_3 aq/EtOH (3:1, v/v). The resulting reaction mixture was stirred at 70 ^{o}C for 3 h, followed by evaporation. The residue was diluted with 50% MeCN/50 mM AcOH/NH_3 buffer or 50 mM AcOH/NH_3 buffer and filtered by membrane filter (0.45 μm). The filtrate solution was dried by lyophilization. The resulting crude mixture was purified by HPLC to give the desired bioconjugates **11** or **12**.

F) Deprotection and cleavage of ACSS in pentapeptide **7** conjugate

Protected pentapeptide conjugates (5 μmol) was added to 5% DCA/CH_2Cl_2 (1 mL) .The resulting mixture was stirred at rt for 5 min, diluted with MeCN (5 mL), neutralized with TEA and collected by vacuum

filtration. The residue was added to CH_2Cl_2 (1 mL) and filtered by membrane filter (0.45 µm). The filtrate solution was evaporated and dried in vacuo. The crude mixture was added to 28% NH_3 aq/EtOH (3:1, v/v). The resulting reaction mixture was stirred at 70 ^oC for 3 h, followed by evaporation. The residue was diluted with 50 mM AcOH/NH_3 buffer and filtered by membrane filter (0.45 µm). The filtrate solution was dried by lyophilization. The resulting crude mixture was TEA 3HF (1 mL) and DMF (1 mL). The resulting mixture was stirred at rt for 24 h, diluted with 50 mM AcOH/NH_3 buffer (1 mL) and purified by C18 column by using 20% MeCN/50 mM AcOH/NH_3 buffer as eluting solvent. The crude mixture was dried by lyophilization. The resulting crude mixture was purified by HPLC to give the desired bioconjugate **8**.

Protected tripeptide conjugate

Crude product as white solid; ^{31}P NMR (242.95 MHz, $CDCl_3$) δ 12.426, 11.722, 9.664, 9.069, -1.570- -3.194, -5.387, -7.959; HRMS $[M + Na]^+$ calculated for $C_{153}H_{221}N_{18}O_{34}P_3Na$ 2971.5256, found 2971.5280.

Deprotected tripeptide conjugate 5

White solid; HRMS $[M - H]^-$ calculated for $C_{49}H_{67}N_{13}O_{24}P_3$ 1314.3640, found.1314.3623.

HPLC spectra are Figure 3 in the Manuscript and Figure S4.

Protected pentapeptide conjugate

Crude product as white solid. ^{31}P NMR (242.95 MHz, $CDCl_3$) δ 9.637. 9.096, -1.651- -2.680; HRMS $[M + Na]^+$ calculated for $C_{214}H_{335}N_{20}O_{39}P_3SiNa$ 3953.3720, found 3953.3739.

Deprotected pentapeptide conjugate 8

White solid. HRMS $[M - H]^-$ calculated for $C_{53}H_{73}N_{15}O_{27}P_3$ 1444.4019, found 1444.4007.

HPLC spectra are Figure S1 and S4.

Protected lipid conjugate

Crude product as white solid. ^{31}P NMR (242.95 MHz, $CDCl_3$) δ 9.967-9.806, 9.402-9.321, -1.104- -1.993; HRMS $[M + Na]^+$ calculated for $C_{145}H_{226}N_{15}O_{28}P_3Na$ 2741.5827, found 2741.5825.

Deprotected lipid conjugate 11

White solid. HRMS $[M - H]^-$ calculated for $C_{56}H_{82}N_{10}O_{20}P_3$ 1307.4925, found 1307.4902.

HPLC spectra are Figure S2 and S4.

Protected sugar conjugate

Crude product as white solid. ^{31}P NMR (242.95 MHz, $CDCl_3$) δ 9.536, 9.051, -1.078- -2.829; HRMS $[M + Na]^+$ calculated for $C_{132}H_{200}N_{15}O_{37}P_3Na$ 2704.3368, found 2704.3371.

Deprotected sugar conjugate 12

White solid. HRMS $[M - H]^-$ calculated for $C_{35}H_{48}N_{10}O_{25}P_3$ 1101.2010, found 1101.1994.

HPLC spectra are Figure S3 and S4.

Suppored decanucleotide 13, Scheme S7

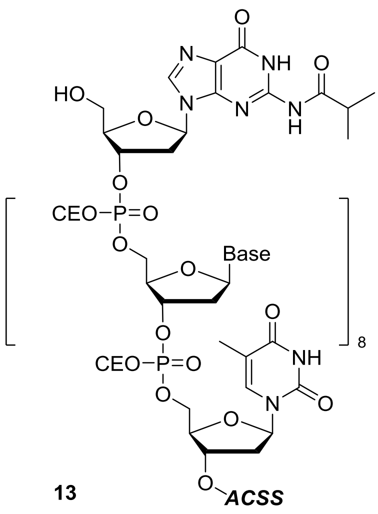

13

Carried out according to the general elongation of oligonucleotide and deprotection of DMTr. 54% yield (0.163 mmol) from **S3** (0.3 mmol) as white solid. ^{31}P NMR (242.95 MHz, $CDCl_3$) δ -2.020; HRMS [M + Na]$^+$ calculated for $C_{243}H_{310}N_{49}O_{71}P_9Na$ 5351.9684 found 5351.9656. Purity was checked by HPLC analysis using a C8 column with H_2O and THF:MeCN=8:2 (70 to 100%, 20 min) as an eluent at flow late of 0.20 mL/min at 50 ^{o}C.

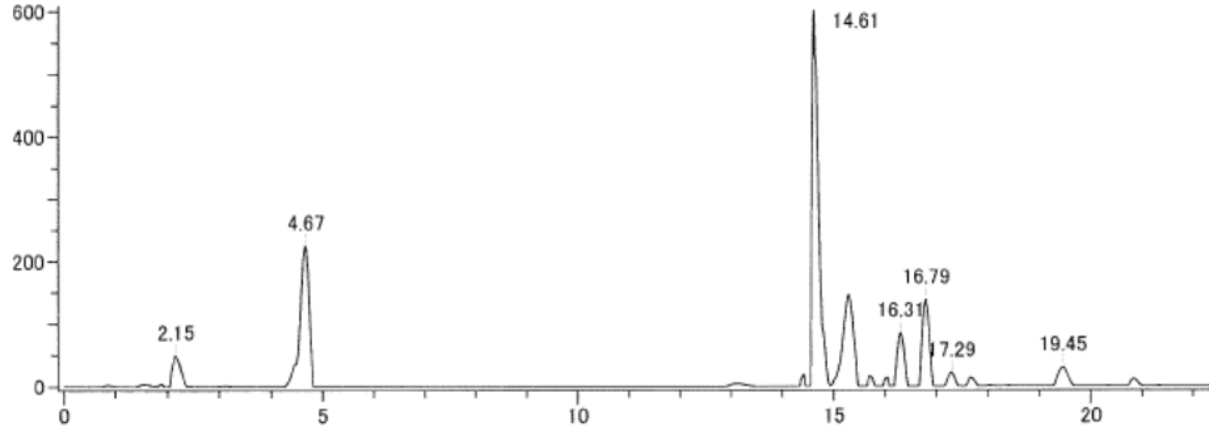

5'-Phosphitylation of the Supported decanucleotide 14, see Scheme 3

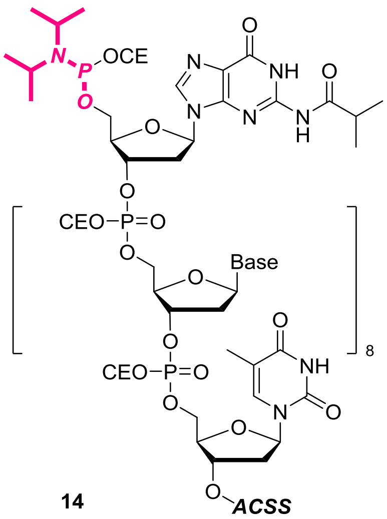

14

Carried out according to the procedure of 5'-phosphitylation of supported trinucletide. 88% yield as white solid. ^{31}P NMR (242.95 MHz, CDCl3) δ 149.839, 149.000, 14.402, -2.003- -2.139.

Bioconjugations and deprotection, see Scheme 4

All protocol was carried out according to the procedure of pentapeptide conjugate (**8**).

Protected pentapeptide conjugate 15

Crude product as white solid. ^{31}P NMR (242.95 MHz, CDCl3) δ 10.048. 9.159, -2.128; HRMS $[M + Na]^+$ calculated for $C_{342}H_{467}N_{55}O_{85}P_{10}SiNa$ 7065.0949, found 7065.0951.

Deprotected pentapeptide conjugate 16

White solid. HRMS $[M - H]^-$ calculated for $C_{121}H_{158}N_{43}O_{67}P_{10}$ 3594.7660, found 3594.7685.

HPLC spectra are Figure 4 in the Manuscript and S4.

(1) Kim. S.; Matsumoto, M.; Chiba, K. *Chem. Eur. J.* **2013**, *19*, 8615 – 8620.

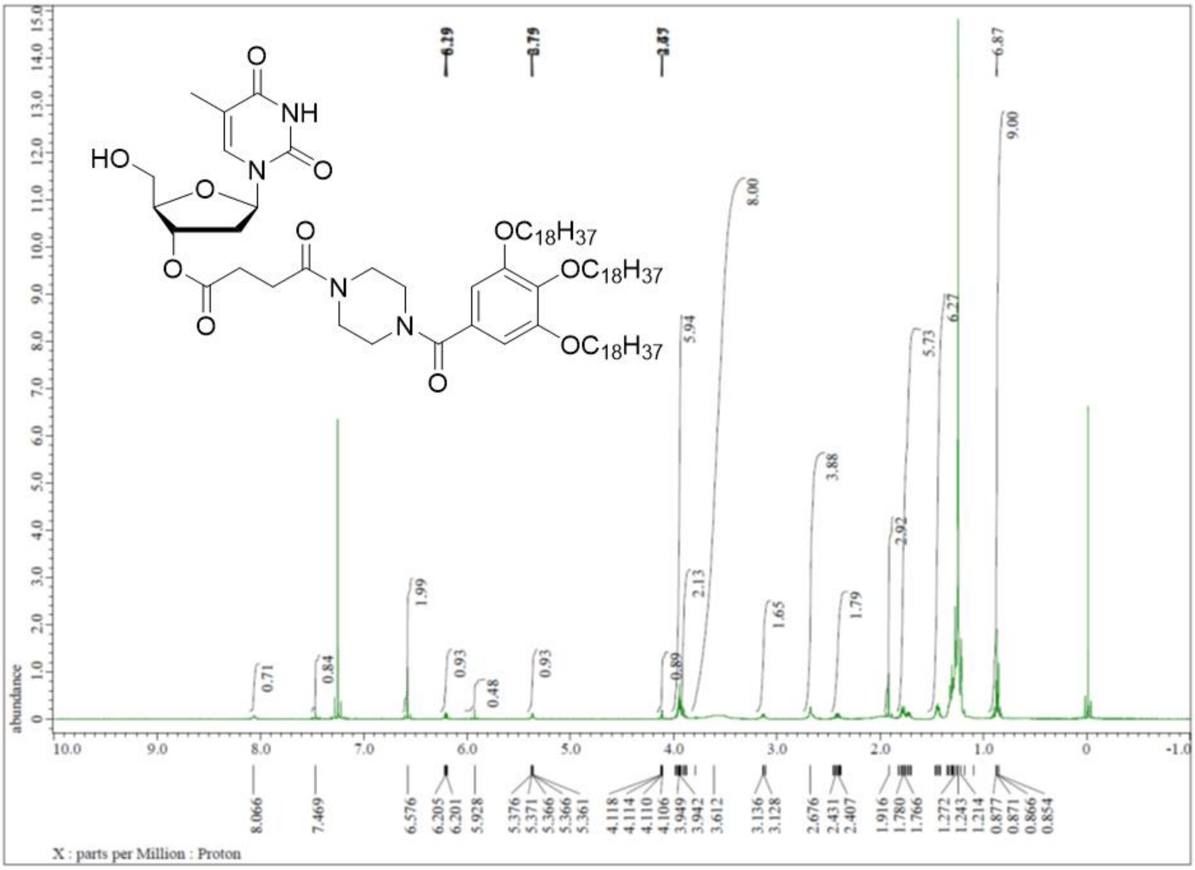

HO
NH
OC18H37
abundance
X : parts per Million : Proton

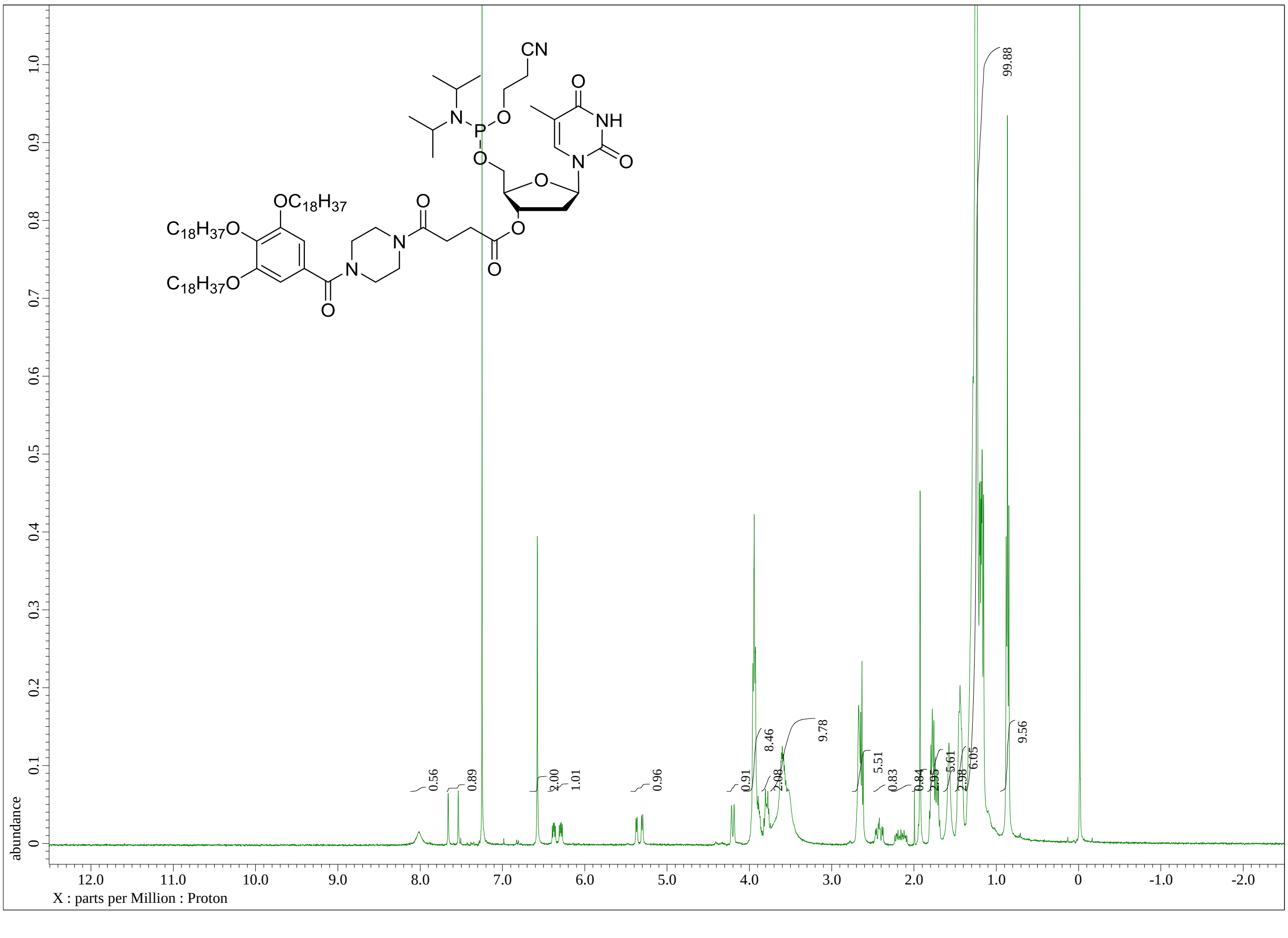

CN
NH
abundance
X : parts per Million : Proton

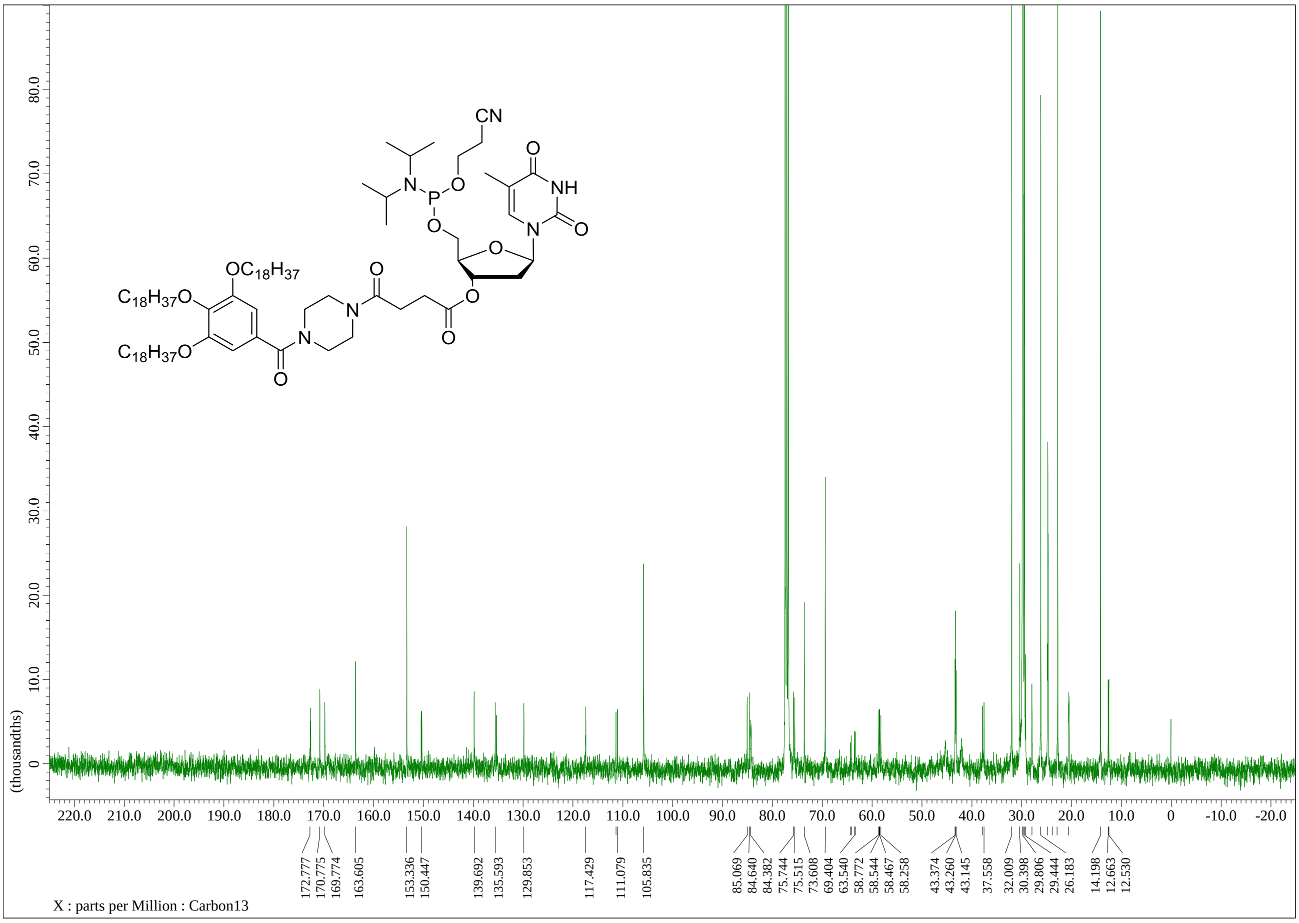

(thousandths)
X : parts per Million : Carbon13
172.777
170.775
169.774
163.605
153.336
150.447
139.692
135.593
129.853
117.429
111.079
105.835
85.069
84.640
84.382
75.744
75.515
73.608
69.404
63.540
58.772
58.544
58.467
58.258
43.374
43.260
43.145
37.558
32.009
30.398
29.806
29.444
26.183
14.198
12.663
12.530

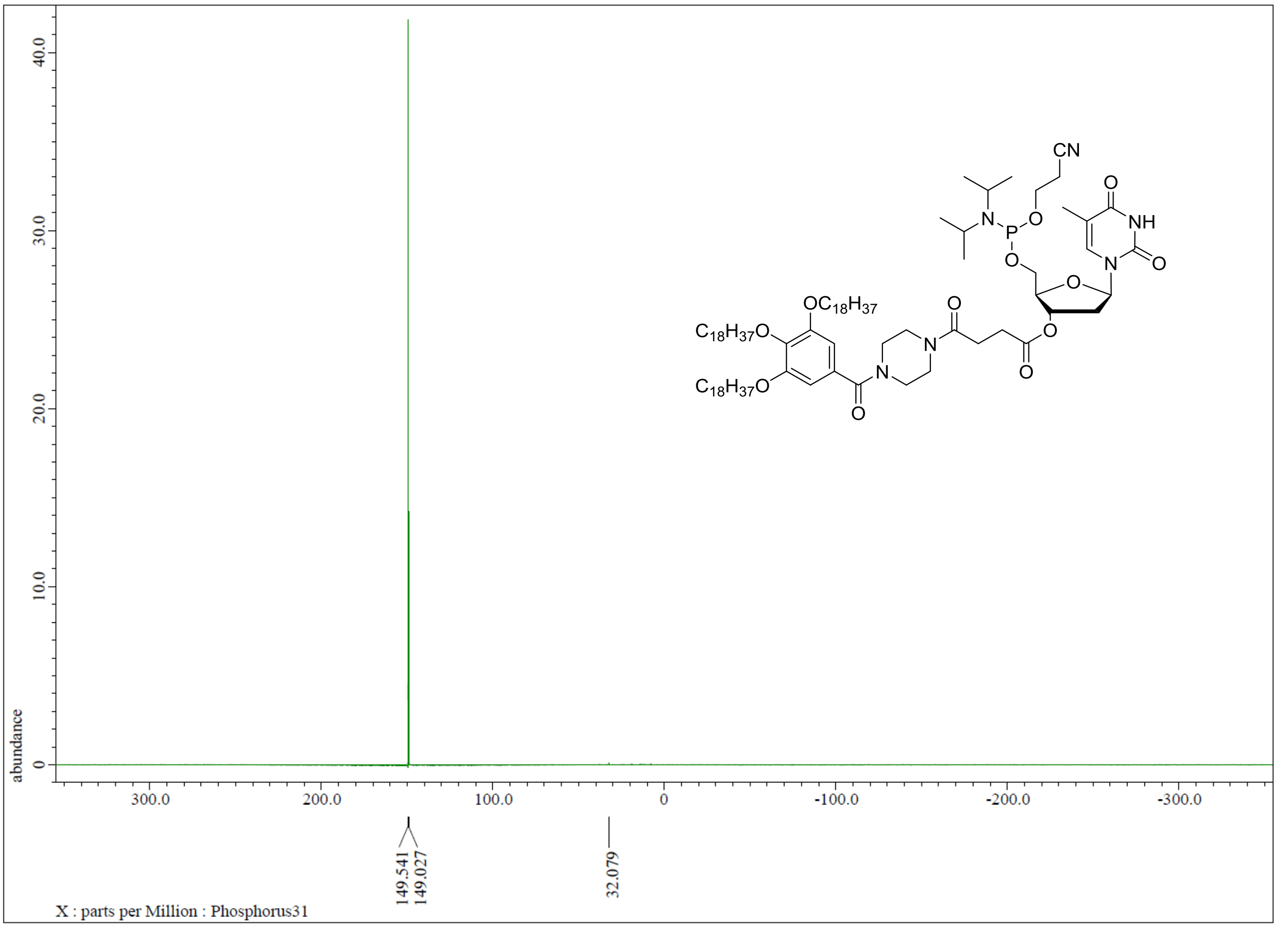

abundance
X : parts per Million : Phosphorus31
149.541
149.027
32.079

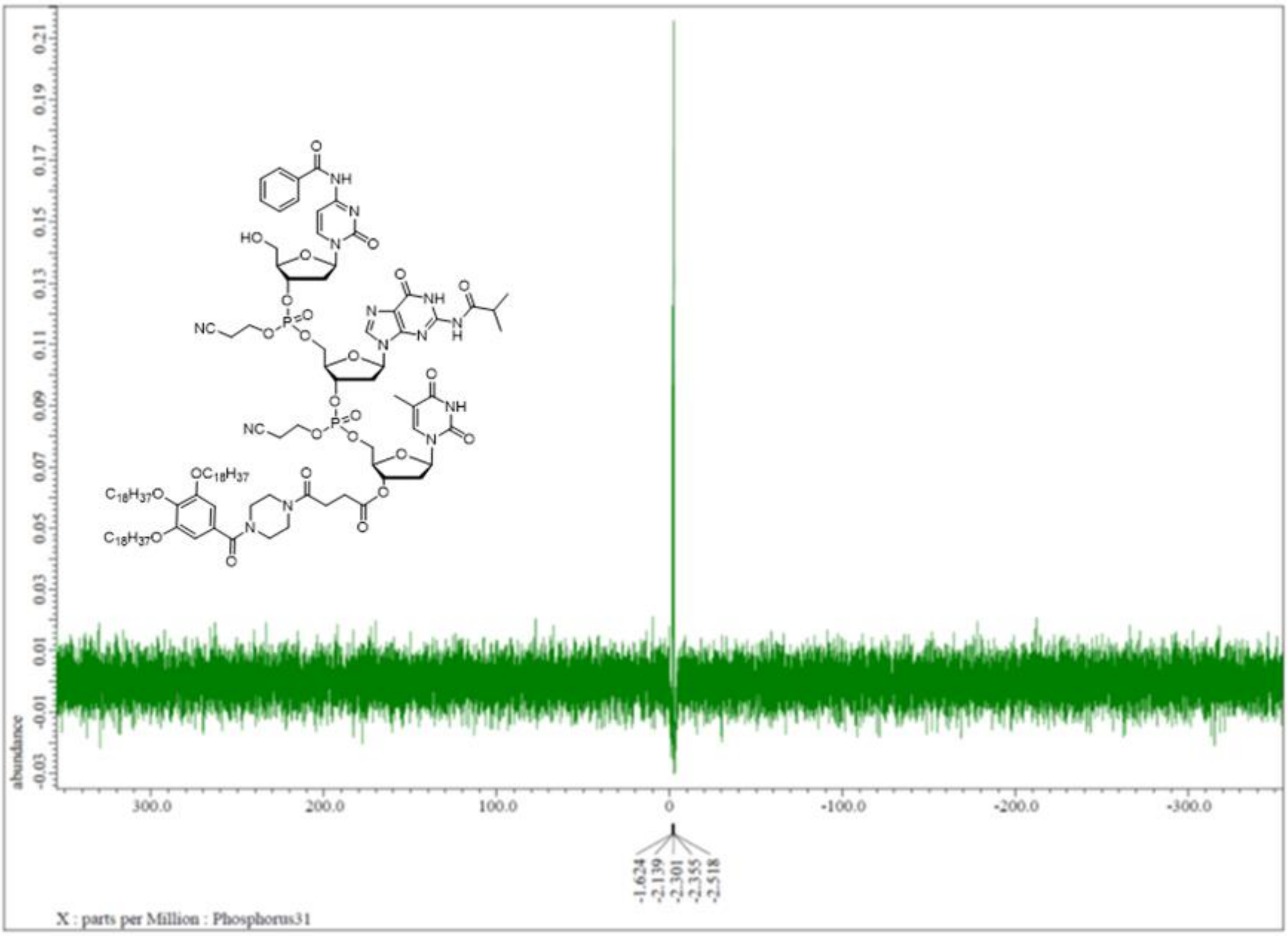
abundance
0.21
0.19
0.17
0.15
0.13
0.11
0.09
0.07
0.05
0.03
0.01
-0.01
-0.03
300.0
200.0
100.0
0
-100.0
-200.0
-300.0
-1.624
-2.139
-2.301
-2.355
-2.518
X : parts per Million : Phosphorus31
HO
NH
NC
NH
N
OC18H37
C18H37O
C18H37O

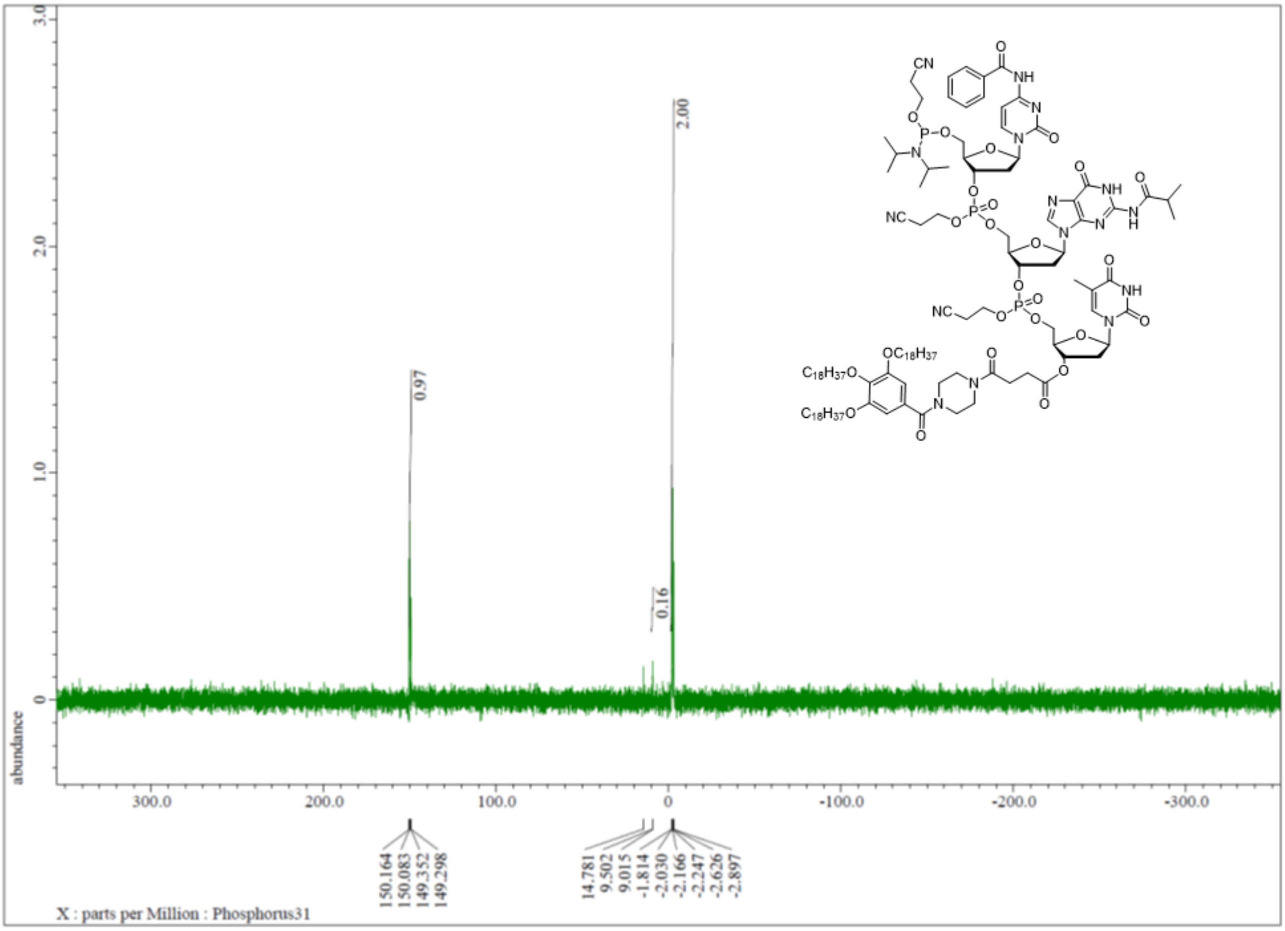
abundance
3.0
2.0
1.0
0
0.97
0.16
2.00
300.0
200.0
100.0
0
-100.0
-200.0
-300.0
150.164
150.083
149.352
149.298
14.781
9.502
9.015
-1.814
-2.030
-2.166
-2.247
-2.626
-2.897
X : parts per Million : Phosphorus31
CN
NH
NC
OC18H37
C18H37O
C18H37O

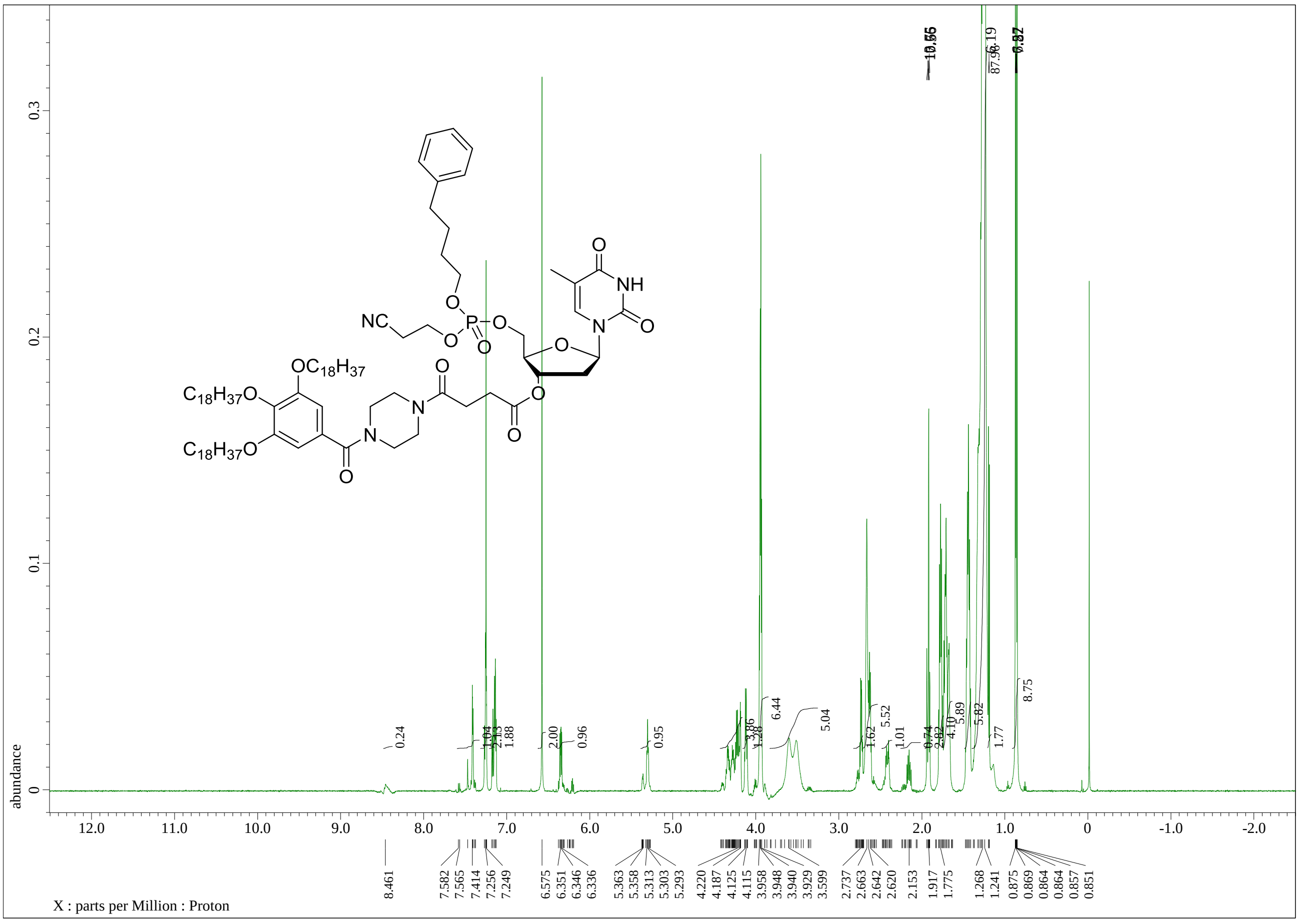

abundance
X : parts per Million : Proton
8.461
7.582
7.565
7.414
7.256
7.249
6.575
6.351
6.346
6.336
5.363
5.358
5.313
5.303
5.293
4.220
4.187
4.125
4.115
3.958
3.948
3.940
3.929
3.599
2.737
2.663
2.642
2.620
2.153
1.917
1.775
1.268
1.241
0.875
0.869
0.864
0.864
0.857
0.851
NC
NH
$OC_{18}H_{37}$
$C_{18}H_{37}O$
$C_{18}H_{37}O$

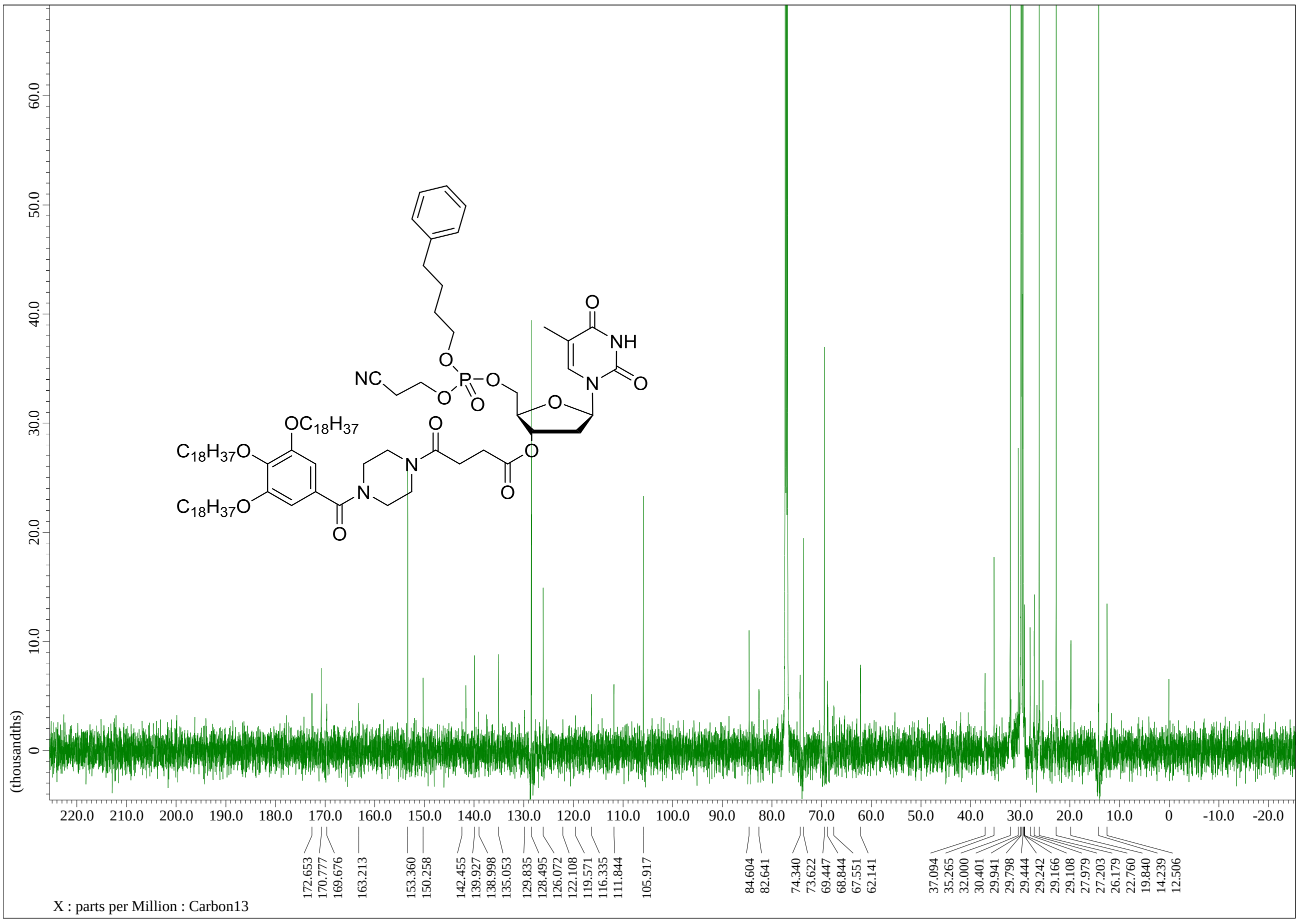

(thousandths)
X : parts per Million : Carbon13
172.653
170.777
169.676
163.213
153.360
150.258
142.455
139.927
138.998
135.053
129.835
128.495
126.072
122.108
119.571
116.335
111.844
105.917
84.604
82.641
74.340
73.622
69.447
68.844
67.551
62.141
37.094
35.265
32.000
30.401
29.941
29.798
29.444
29.242
29.166
29.108
27.979
27.203
26.179
22.760
19.840
14.239
12.506
NC
NH
$OC_{18}H_{37}$
$C_{18}H_{37}O$
$C_{18}H_{37}O$

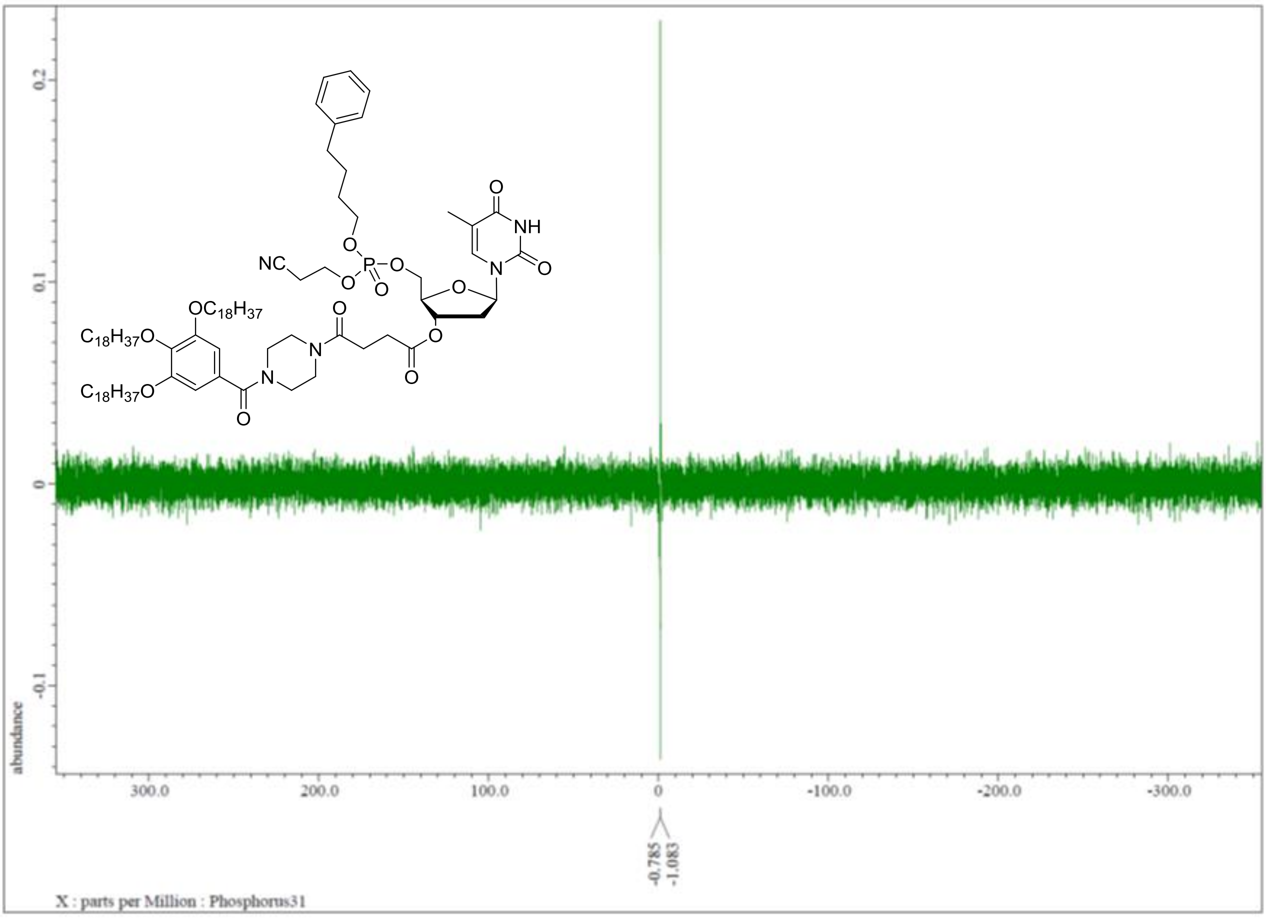

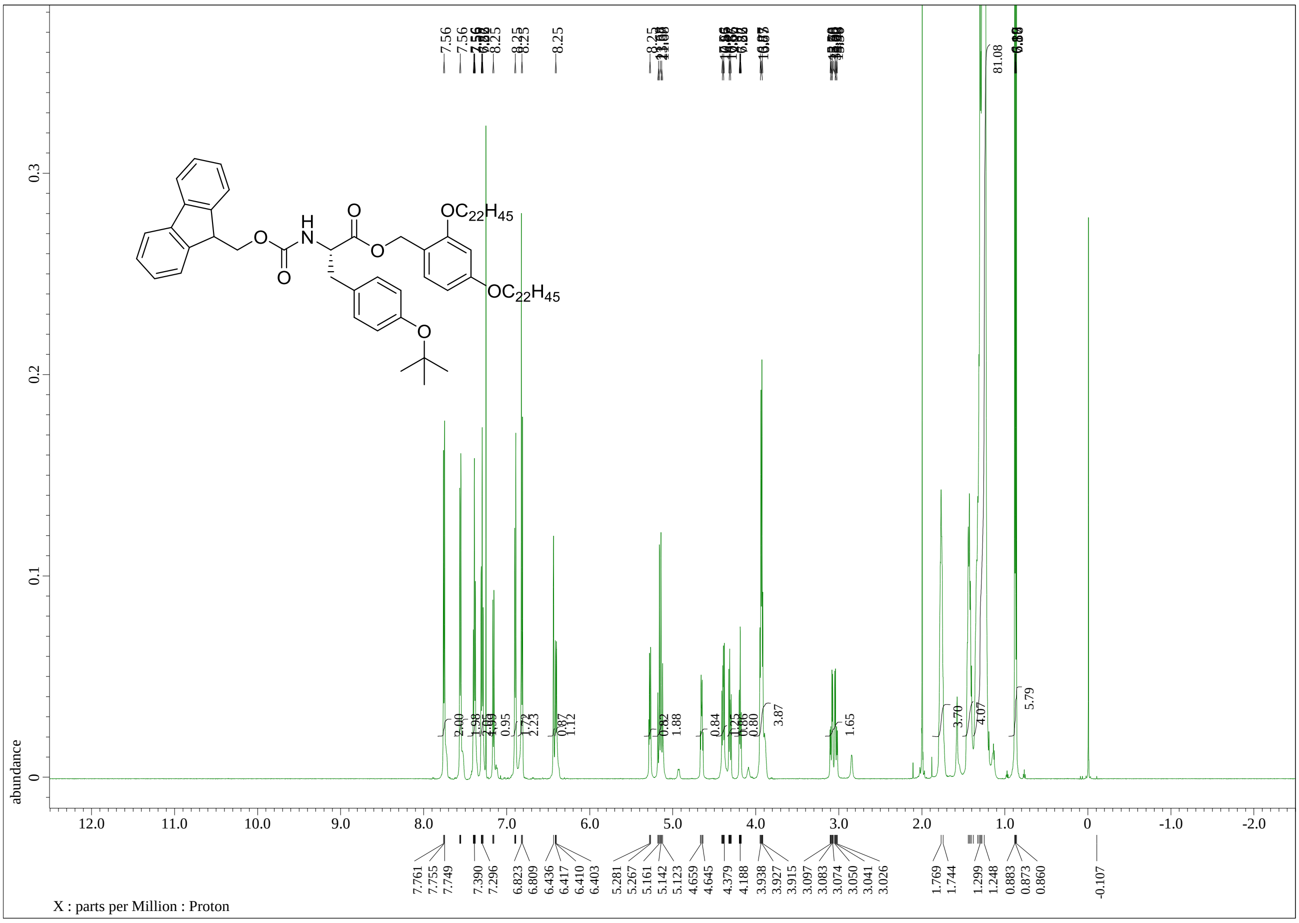

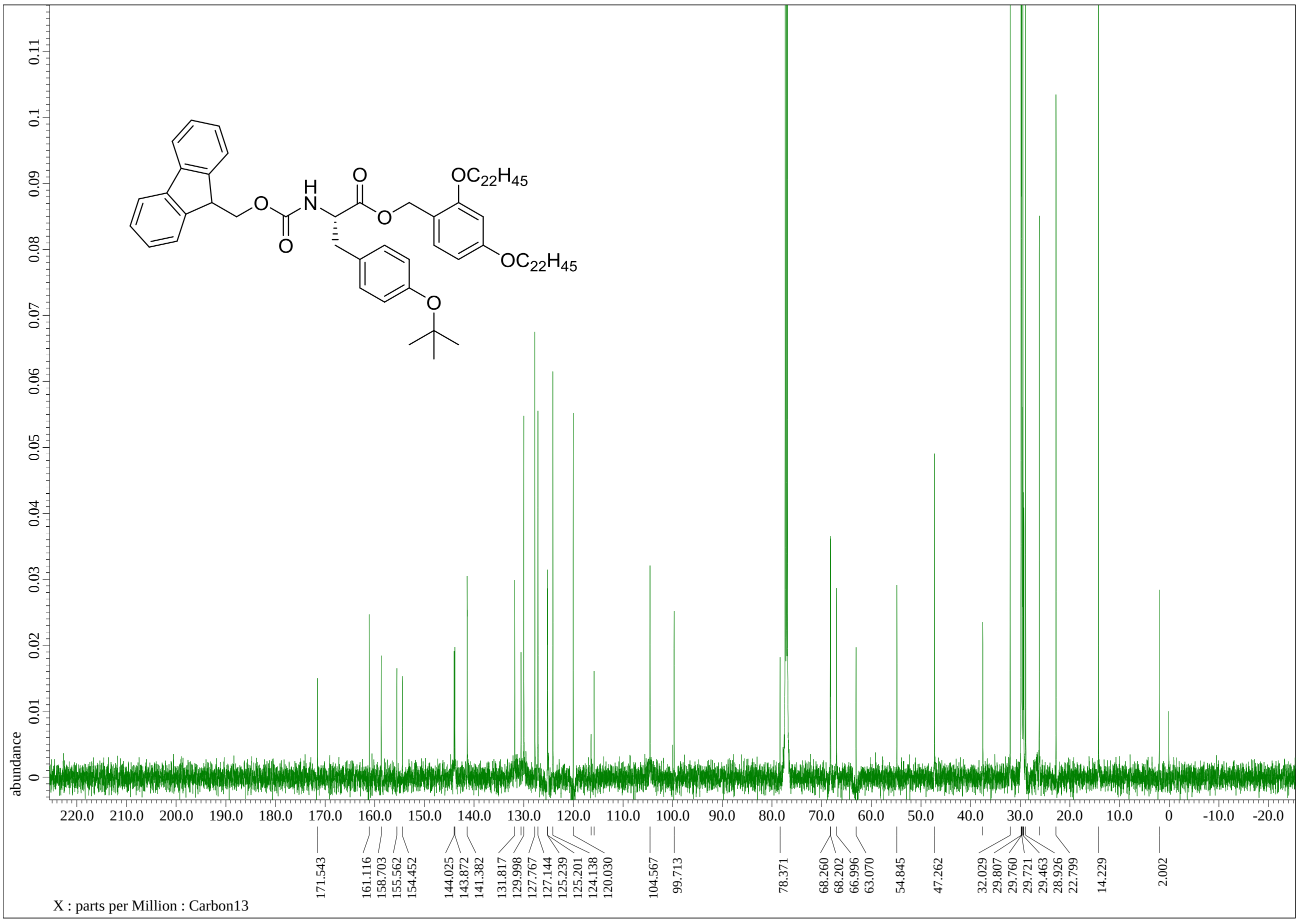

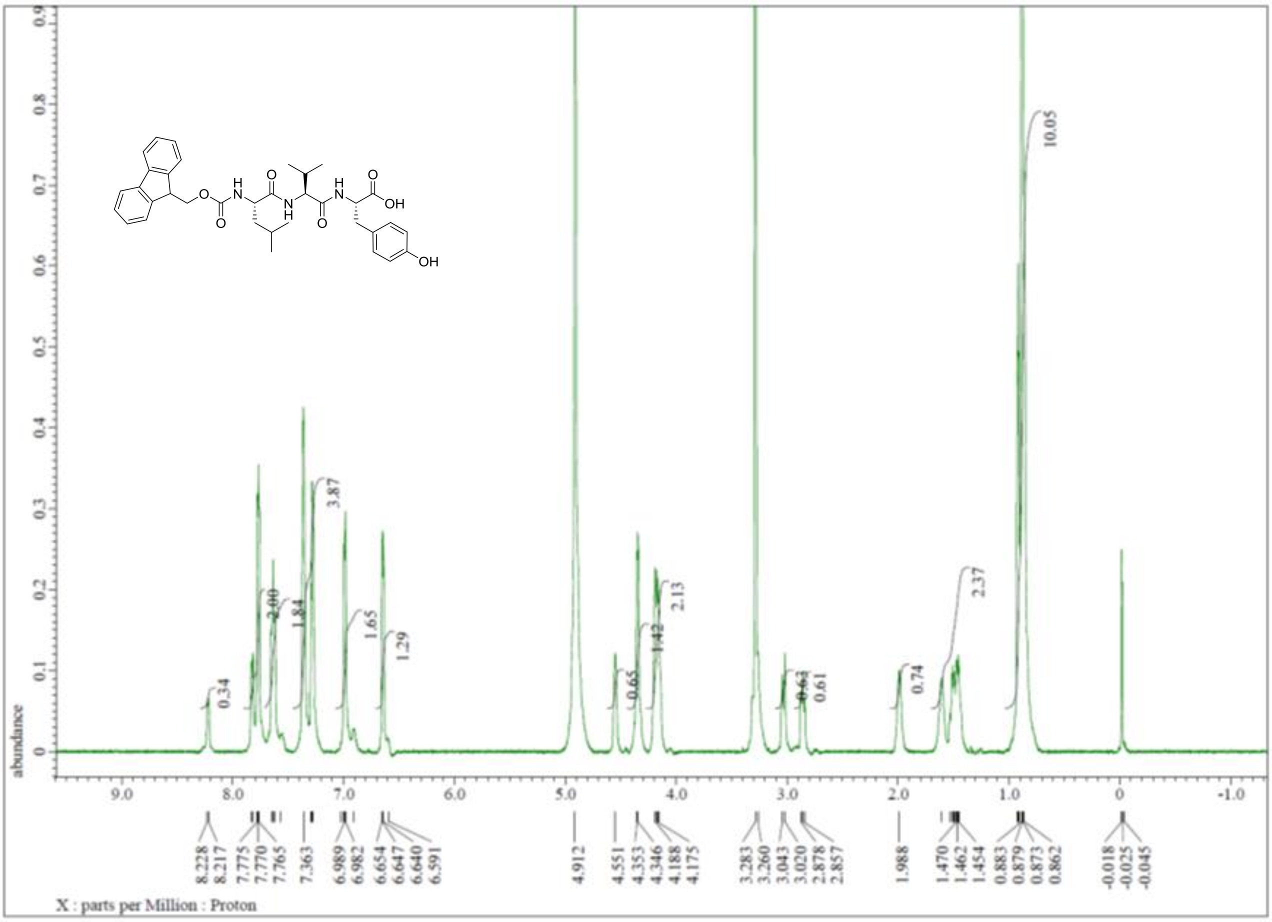

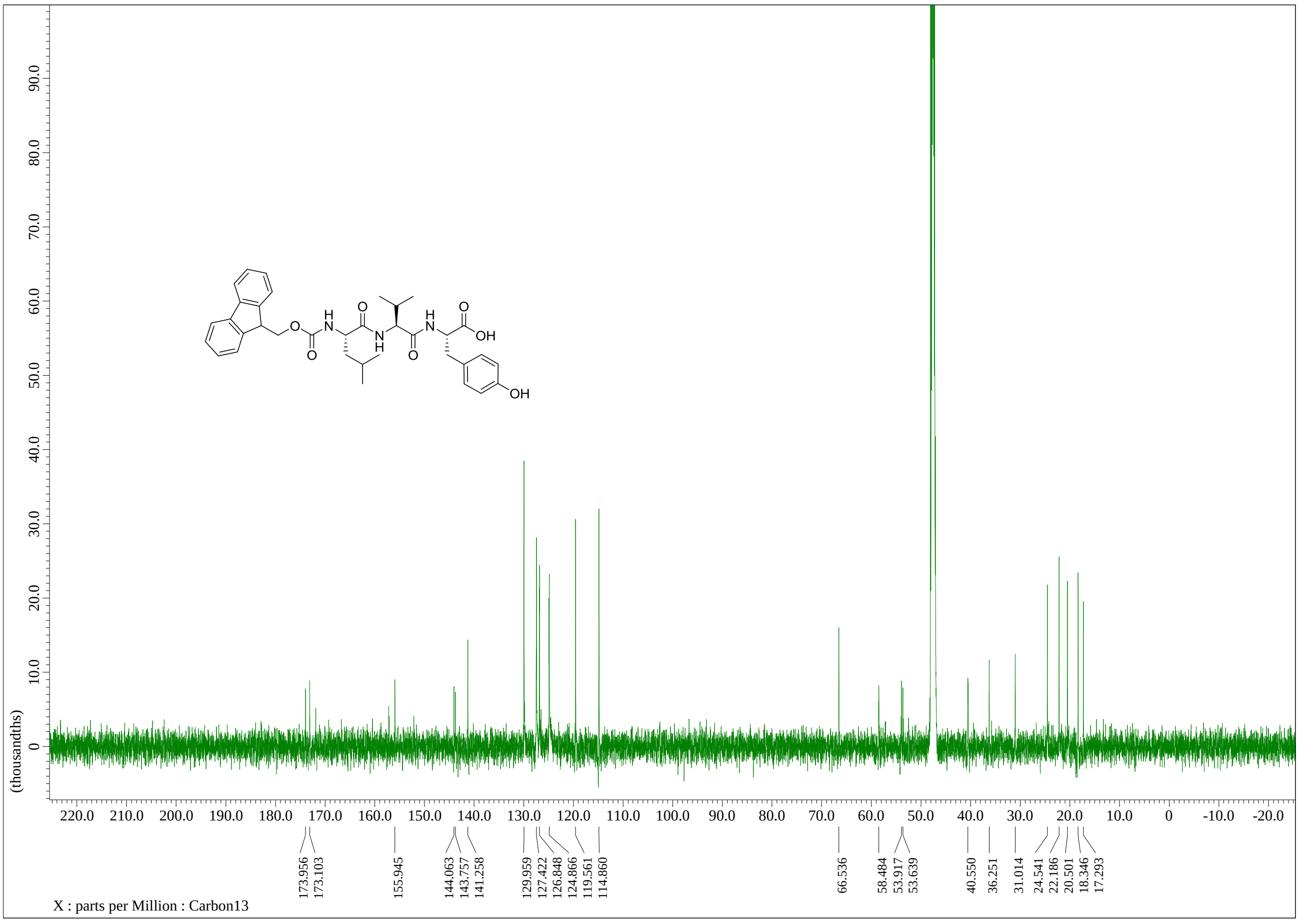

X : parts per Million : Carbon13
(thousandths)
173.956
173.103
155.945
144.063
143.757
141.258
129.959
127.422
126.848
124.866
119.561
114.860
66.536
58.484
53.917
53.639
40.550
36.251
31.014
24.541
22.186
20.501
18.346
17.293

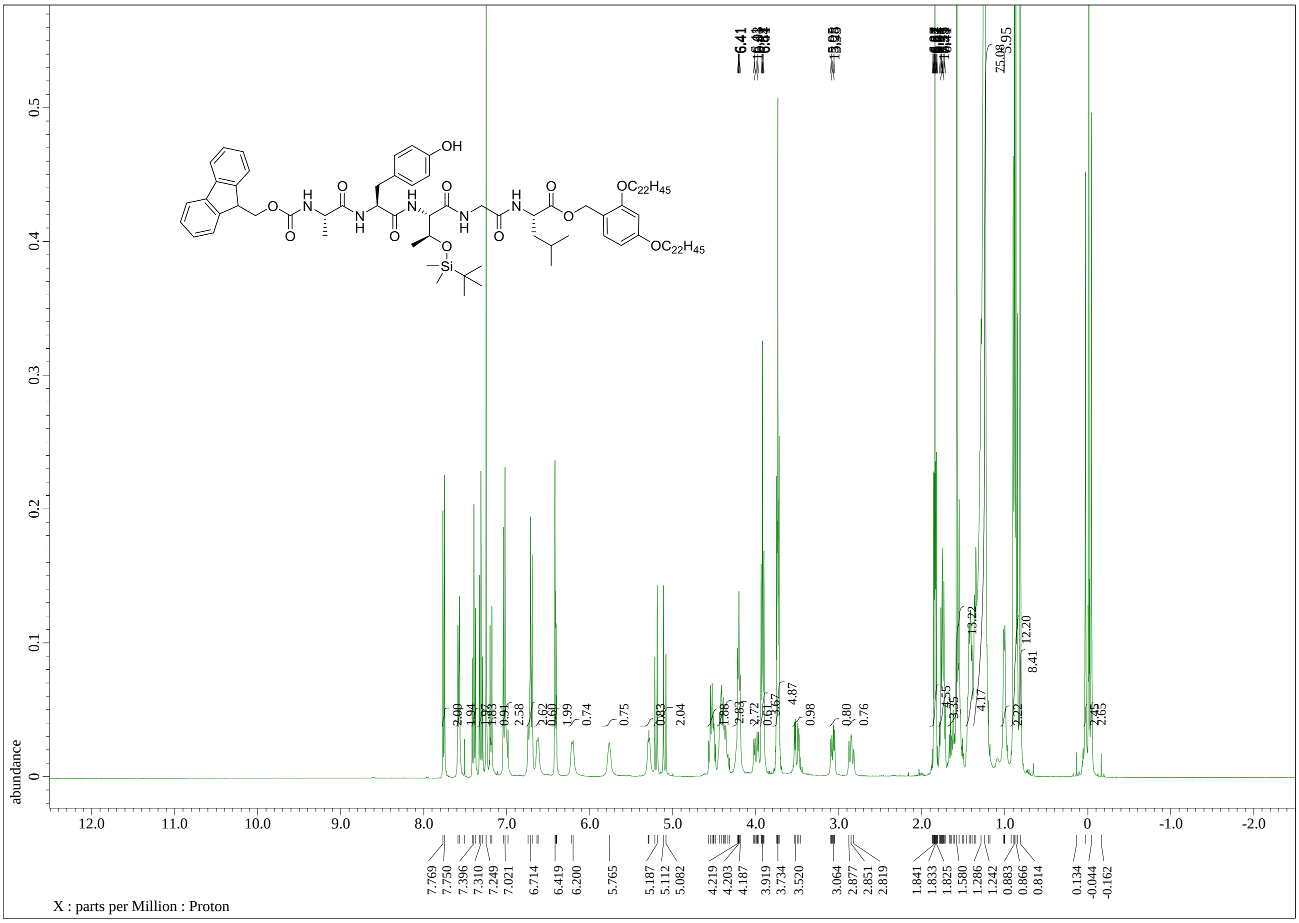

X : parts per Million : Proton
abundance
7.769
7.750
7.396
7.310
7.249
7.021
6.714
6.419
6.200
5.765
5.187
5.112
5.082
4.219
4.203
4.187
3.919
3.734
3.520
3.064
2.877
2.851
2.819
1.841
1.833
1.825
1.580
1.286
1.242
0.883
0.866
0.814
0.134
-0.044
-0.162

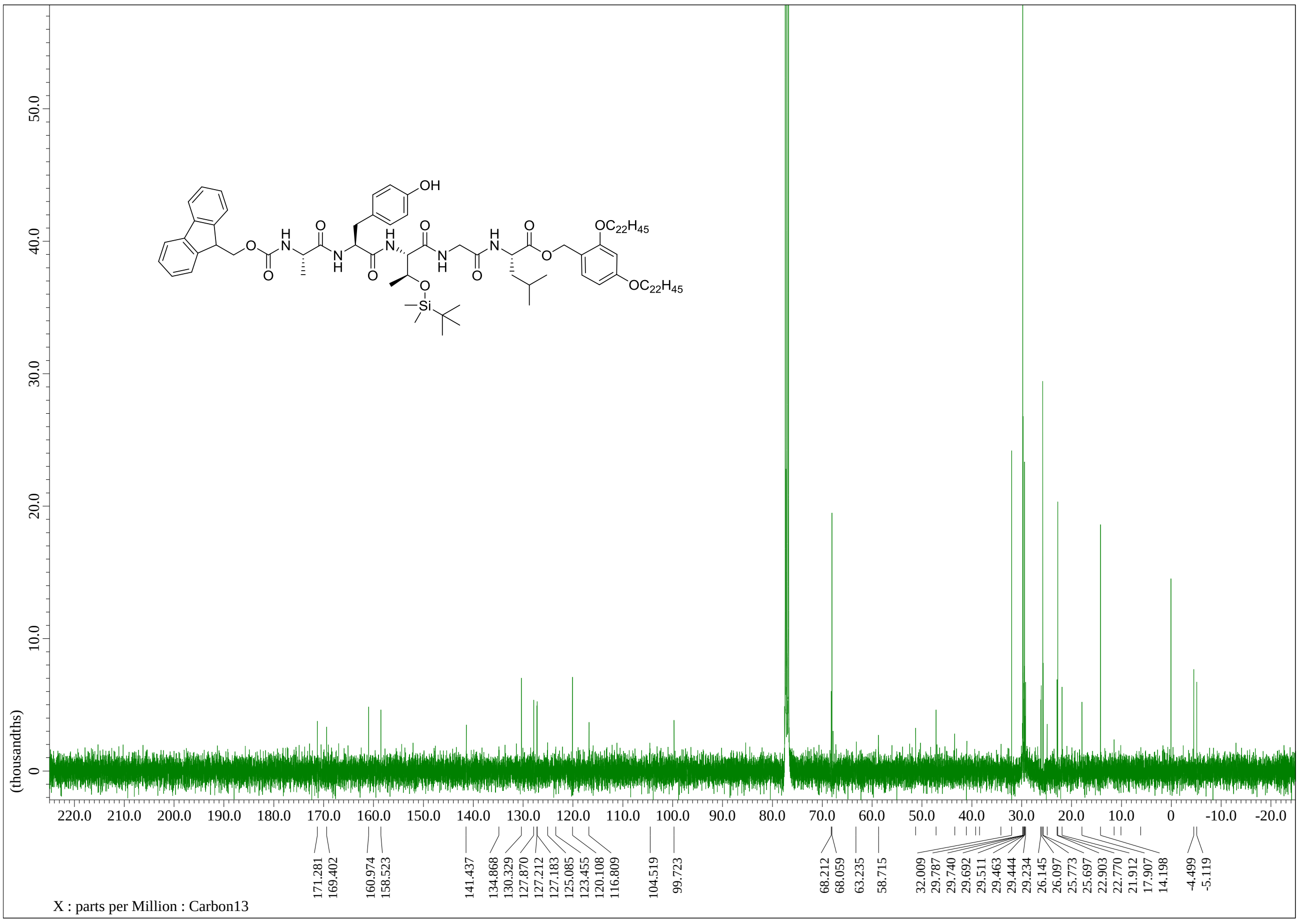

(thousandths)
50.0
40.0
30.0
20.0
10.0
0
220.0 210.0 200.0 190.0 180.0 170.0 160.0 150.0 140.0 130.0 120.0 110.0 100.0 90.0 80.0 70.0 60.0 50.0 40.0 30.0 20.0 10.0 0 -10.0 -20.0
171.281 169.402 160.974 158.523 141.437 134.868 130.329 127.870 127.212 127.183 125.085 123.455 120.108 116.809 104.519 99.723 68.212 68.059 63.235 58.715 32.009 29.787 29.740 29.692 29.511 29.463 29.444 29.234 26.145 26.097 25.773 25.697 22.903 22.770 21.912 17.907 14.198 -4.499 -5.119
X : parts per Million : Carbon13
OH
OC22H45
OC22H45
Si

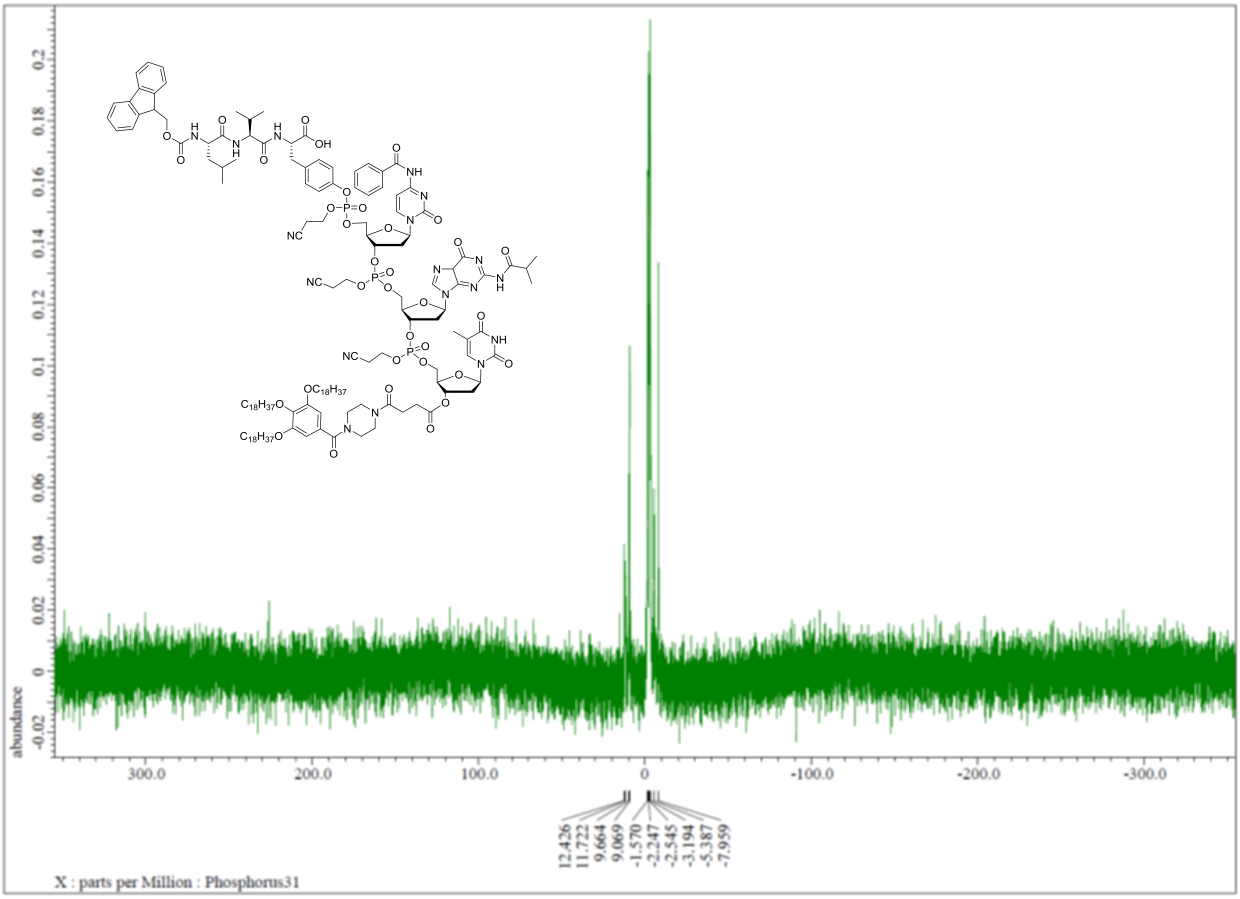

abundance
0.2
0.18
0.16
0.14
0.12
0.1
0.08
0.06
0.04
0.02
0
-0.02
300.0 200.0 100.0 0 -100.0 -200.0 -300.0
12.426 11.722 9.664 9.069 -1.570 -2.247 -2.545 -3.194 -5.387 -7.959
X : parts per Million : Phosphorus31
NC
OC18H37
C18H37O
C18H37O

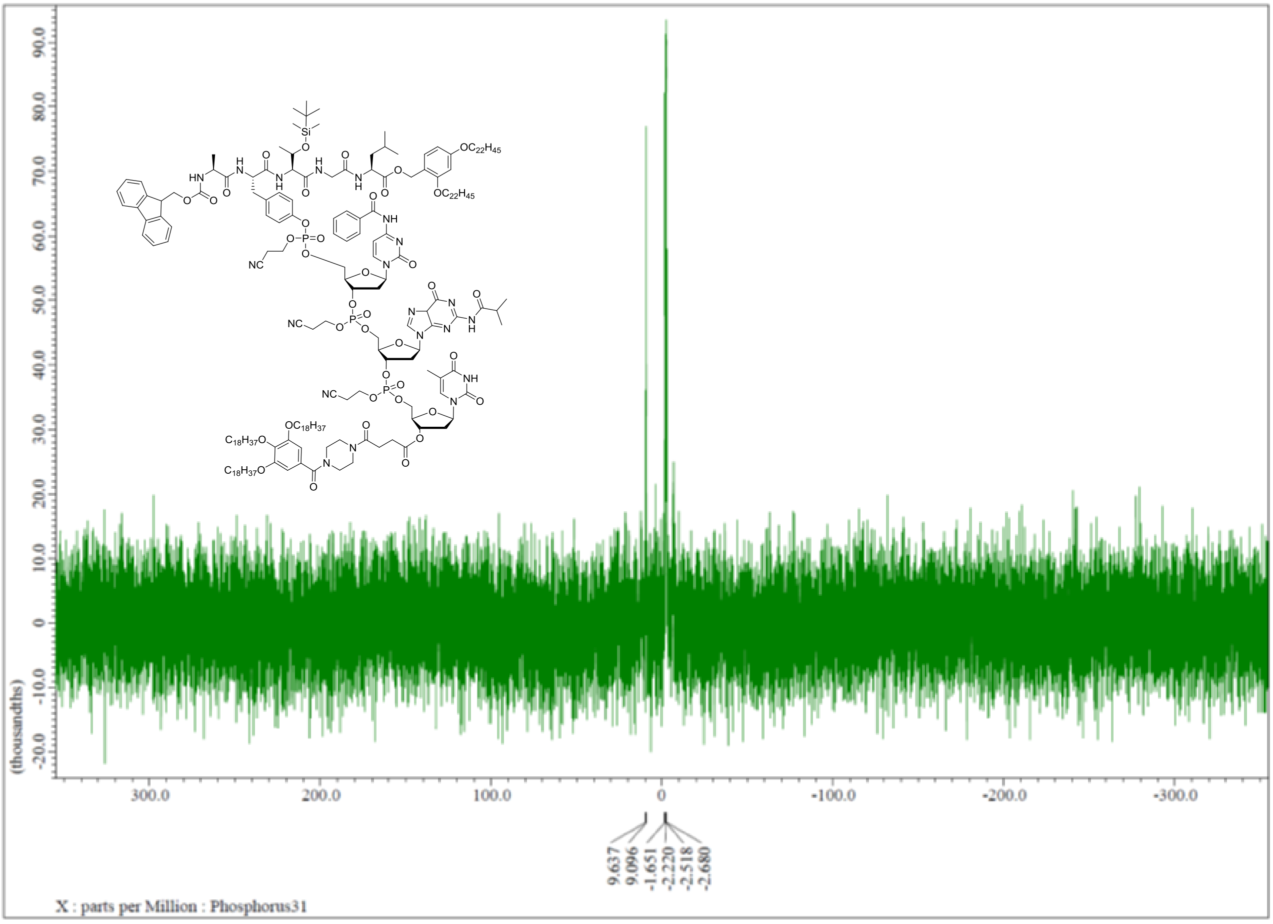

X : parts per Million : Phosphorus31
(thousandths)
9.637
9.096
-1.651
-2.220
-2.518
-2.680

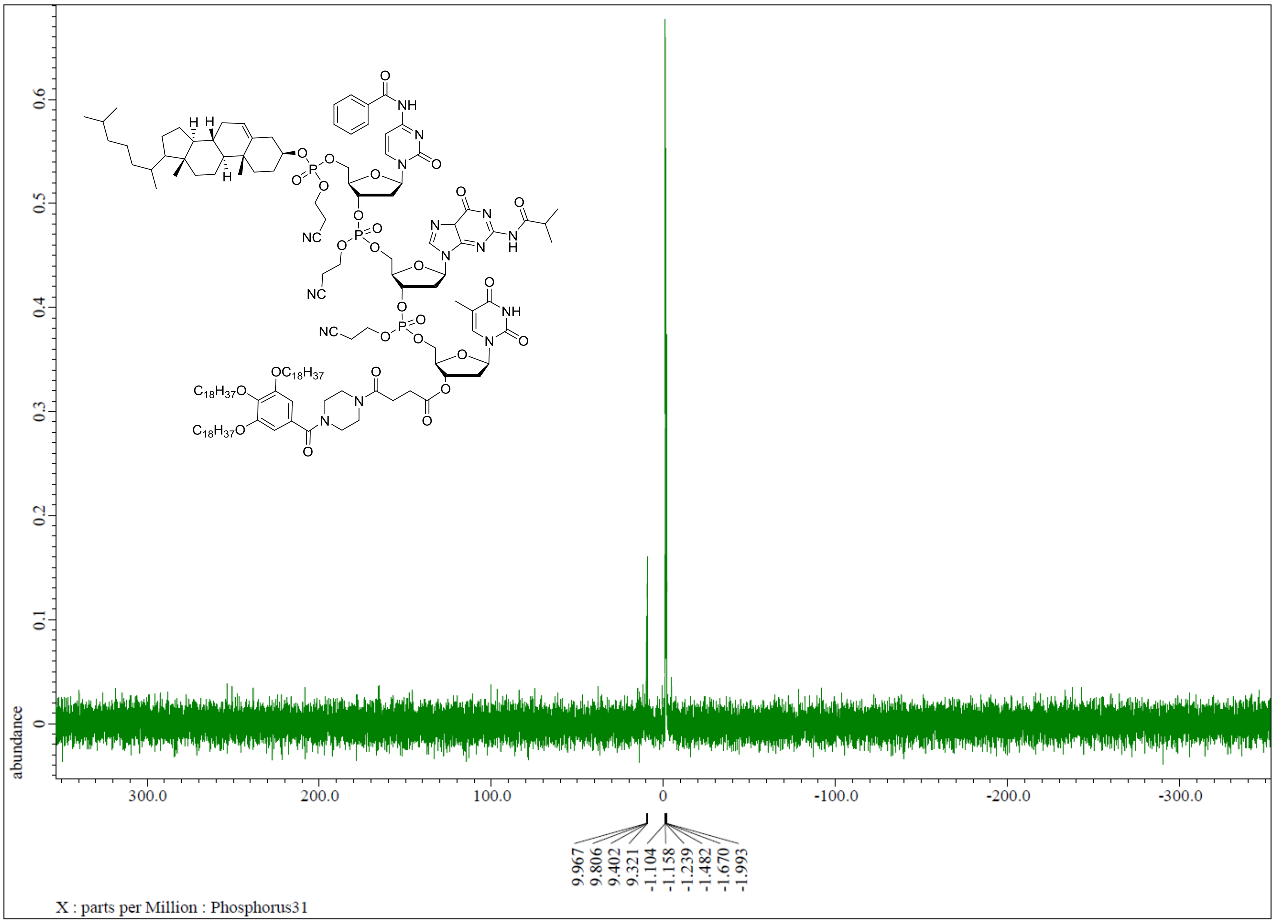

X : parts per Million : Phosphorus31
abundance
9.967
9.806
9.402
9.321
-1.104
-1.158
-1.239
-1.482
-1.670
-1.993

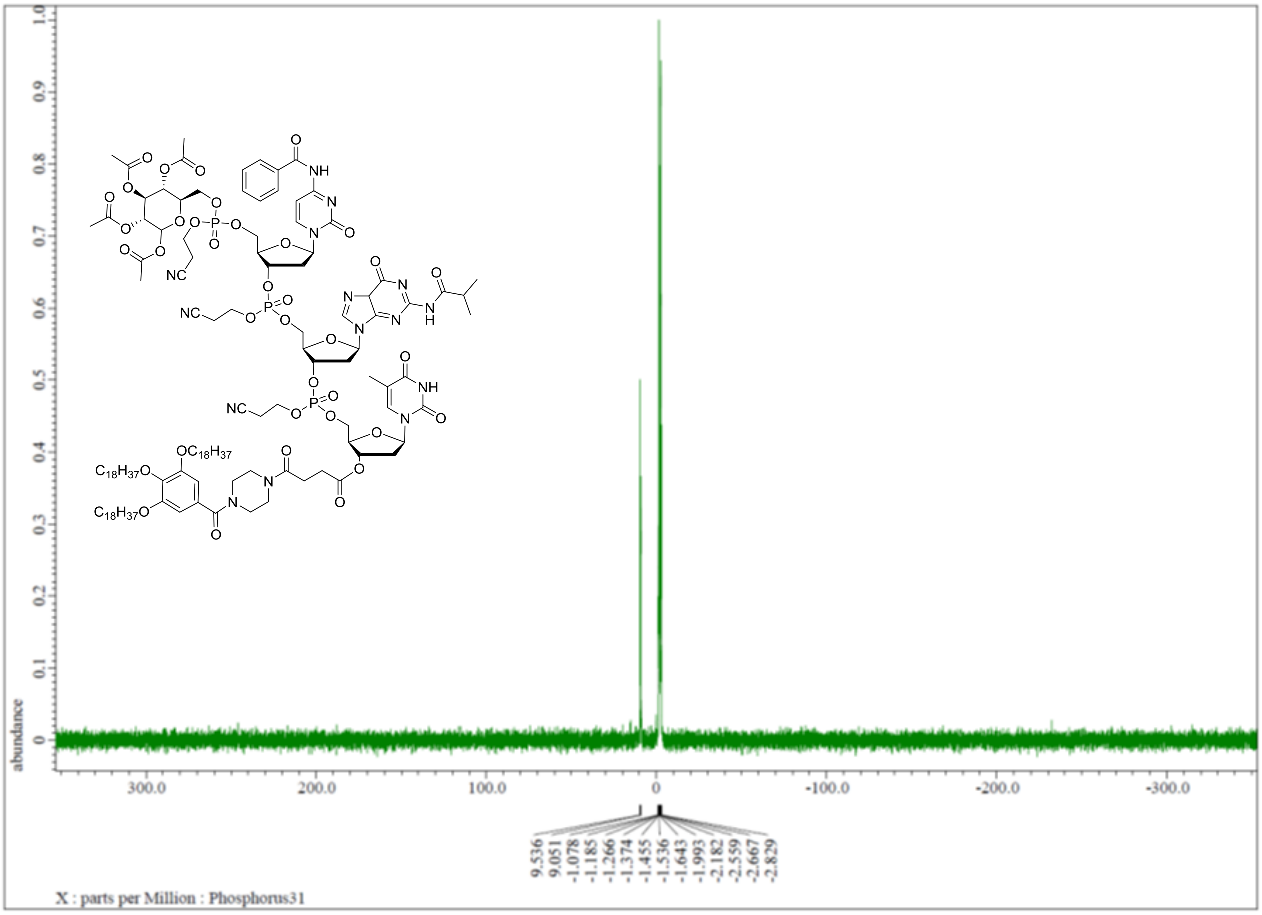

abundance
300.0
200.0
100.0
0
-100.0
-200.0
-300.0
9.536
9.051
-1.078
-1.185
-1.266
-1.374
-1.455
-1.536
-1.643
-1.993
-2.182
-2.559
-2.667
-2.829
X : parts per Million : Phosphorus31

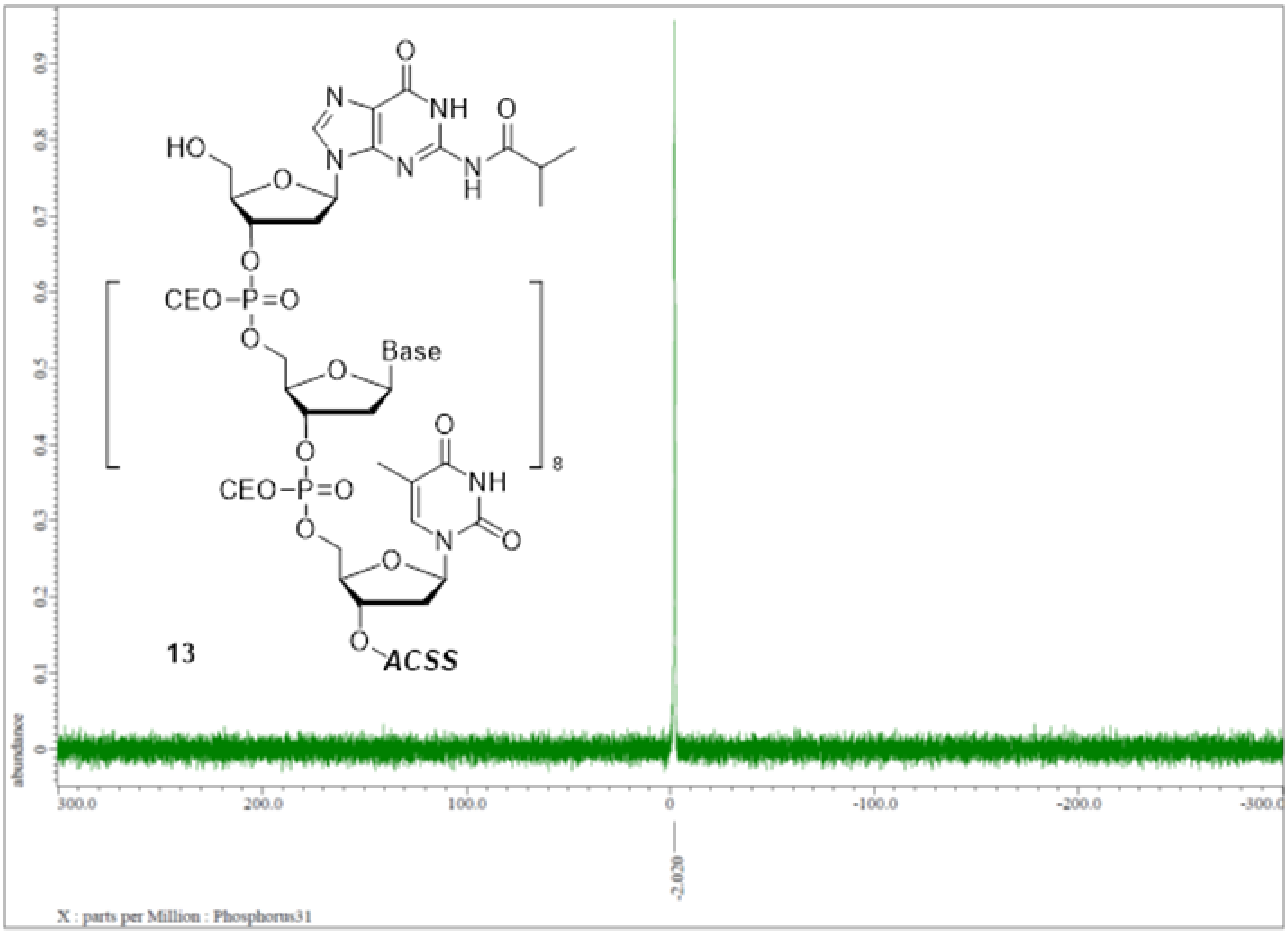

abundance
CEO-P=O
Base
13
ACSS
8
300.0
200.0
100.0
0
-100.0
-200.0
-2.020
X : parts per Million : Phosphorus31

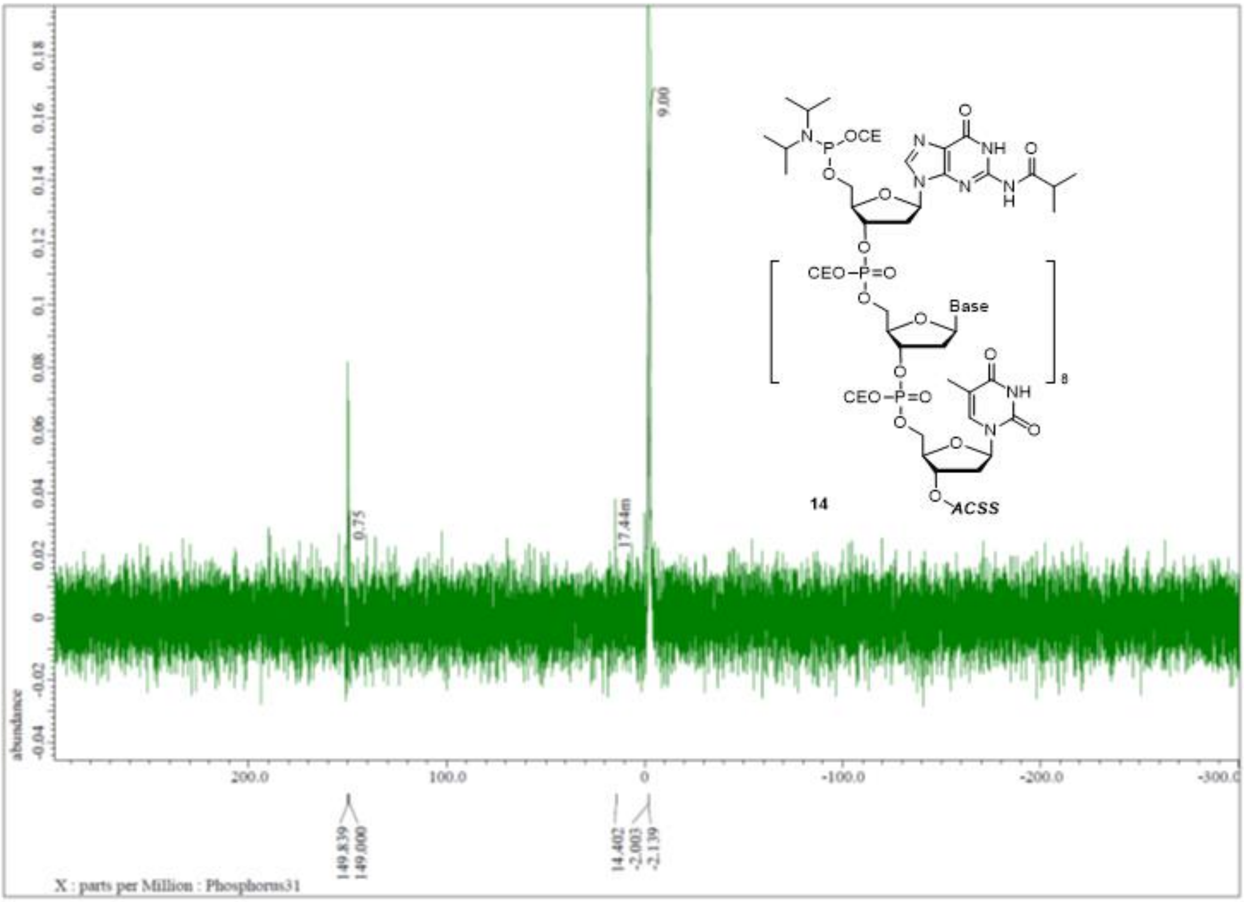

14
CEO–P=O
Base
OCE
ACSS
8
abundance
X : parts per Million : Phosphorus31
149.839
149.000
14.402
-2.003
-2.139
9.00
0.75
17.44m

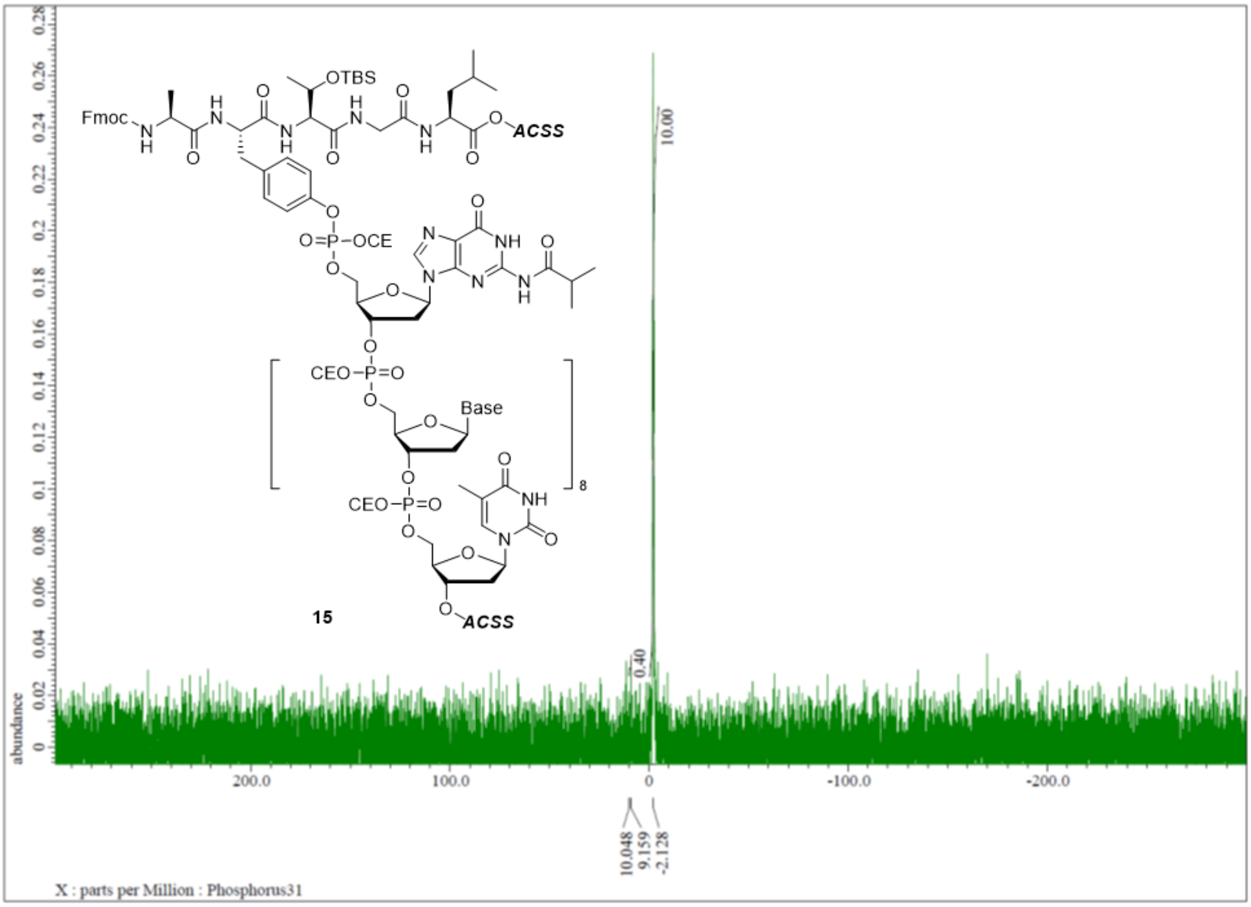

15
Fmoc
OTBS
O=P–OCE
CEO–P=O
Base
ACSS
8
abundance
X : parts per Million : Phosphorus31
10.048
9.159
-2.128
10.00
0.40
